# Communication inequalities and health disparities among vulnerable groups during the COVID-19 pandemic - a scoping review of qualitative and quantitative evidence

**DOI:** 10.1186/s12889-023-15295-6

**Published:** 2023-03-06

**Authors:** Clara Häfliger, Nicola Diviani, Sara Rubinelli

**Affiliations:** 1 Swiss Paraplegic Research, Guido A. Zäch Strasse 4, Nottwil, Lucerne, 6207 Switzerland; 2 Faculty of Health Sciences and Medicine, University of Lucerne, Frohburgstrasse 3, Lucerne, 6002 Switzerland

**Keywords:** Communication inequalities, Health disparities, COVID-19, Social determinants, Vulnerable groups, Public health crises, Structural Influence Model

## Abstract

**Background:**

The COVID-19 pandemic has exacerbated health disparities in vulnerable groups (e.g., increased infection, hospitalization, and mortality rates in people with lower income, lower education, or ethnic minorities). Communication inequalities can act as mediating factors in this relationship. Understanding this link is vital to prevent communication inequalities and health disparities in public health crises. This study aims to map and summarize the current literature on communication inequalities linked with health disparities (CIHD) in vulnerable groups during the COVID-19 pandemic and to identify research gaps.

**Methods:**

A scoping review of quantitative and qualitative evidence was conducted. The literature search followed the guidelines of PRISMA extension for scoping reviews and was performed on PubMed and PsycInfo. Findings were summarized using a conceptual framework based on the Structural Influence Model by Viswanath et al.

**Results:**

The search yielded 92 studies, mainly assessing low education as a social determinant and knowledge as an indicator for communication inequalities. CIHD in vulnerable groups were identified in 45 studies. The association of low education with insufficient knowledge and inadequate preventive behavior was the most frequently observed. Other studies only found part of the link: communication inequalities (*n* = 25) or health disparities (*n* = 5). In 17 studies, neither inequalities nor disparities were found.

**Conclusions:**

This review supports the findings of studies on past public health crises. Public health institutions should specifically target their communication to people with low education to reduce communication inequalities. More research about CIHD is needed on groups with migrant status, financial hardship, not speaking the language in the country of residence, sexual minorities, and living in deprived neighborhoods. Future research should also assess communication input factors to derive specific communication strategies for public health institutions to overcome CIHD in public health crises.

**Supplementary Information:**

The online version contains supplementary material available at 10.1186/s12889-023-15295-6.

## Background

Environmental conditions in which people were born, live, and age have a tremendous impact on the health of people all over the globe [1–3]. Defined as the social determinants of health, they are considered a public health issue by the World Health Organization (WHO) [2] and Healthy People 2030 [1]. Social determinants of health contribute substantially to health differences related to socioeconomic disadvantage, referred to as health disparities [1, 4, 5].

Public health crises tend to exacerbate health disparities [6]. O'Sullivan and Bourgoin, for example, found in a review, that lower financial resources during a pandemic are associated with lower access to supportive care and poorer living conditions (e.g. crowded housing) with an increased risk of an infection during a pandemic [6]. Other social determinants found for an increased health risk during a pandemic were ethnicity, language, culture, health literacy, elderly, and employment status [6]. Recent evidence confirms that this is equally true for the current COVID-19 pandemic [7–22]. Emerging examples during the COVID-19 pandemic are increased mortality [9], hospitalizations [10], infection rates [7], and lower preventive behavior [14] in socially vulnerable population groups (Table 1). Therefore, it is essential to consider vulnerability not only based on an increased risk for medical complications or mortality from infection [6]. It is also vital to acknowledge the social gradient of risk for socially vulnerable groups to experience adverse health outcomes [6]. Identifying and understanding the underlying mechanisms is essential to addressing such health disparities [4].

**Table 1:** Identified social determinants of health disparities in the COVID-19 pandemic

Social determinants	Health disparities	Reference
Low income	Increased infection rate	[7, 8]
	Increased death rate	[9]
	Increased mental health problems	[11]
	Lower preventive behavior	[14]
Low education	Increased infection rate	[7, 8]
	Increased death rate	[10]
	Increased hospitalization rate	[10]
	Lower preventive behavior	[14]
Deprived neighborhood	Increased infection rate	[7]
	Lower vaccination rate	[13]
Unemployment	Increased death rate	[18]
Financial hardship	Increased infection rate	[7]
	Increased mental health problems	[22]
Ethnic minority	Increased infection rate	[7, 12, 20]
	Increased death rate	[9, 10]
	Increased hospitalization rate	[10]
	Lower vaccination rate	[13]
	Lower testing behavior	[16]
Chronic condition	Increased mental health problems	[19]
Migrant status	Increased infection rate	[15]
	Increased mental health problems	[17]
Sexual minority	Increased mental health problems	[21]
Older age (> 60 years)	Increased infection rate	[7, 20]
Not speaking the language of the country of residence	Increased infection rate	[7]

One explanation is provided in the Structural Influence Model (SIM) by Viswanath et al., using communication as a mediating factor between social determinants of health and health outcomes [23]. The SIM states that social determinants influence communication outcomes [23–25]. This may result in communication inequalities, defined as "inequalities in individual or group-specific exposure to public health communication messages, and in the capacity to access, process, and act upon the information received…[26]". The model is well-supported by evidence of different adverse communication outcomes in vulnerable groups [24, 27–30]. Communication inequalities can lead to adverse health outcomes and thus, reinforce health disparities [23–25]. Low education and low income, for example, are found to be associated with increased smoking behavior. Health media consumption and risk perception related to smoking were mediating factors in this association [24].

In public health emergencies, where people must be informed about risks and preventive behaviors, communication inequalities are likely to occur [26, 31]. Established examples are Hurricane Katrina, where unemployed people were less exposed to evacuation messages [32], or the MERS outbreak, where people with lower education showed lower information-seeking behavior as well as lower preventive behavior [33, 34]. Similar findings are available for the H1N1 pandemic [26], and, most recently, the COVID-19 pandemic [35]. Despite the hope that modern communication technologies, including social media, might help democratize information, evidence of communication inequalities emerged already in the early stages of the COVID-19 pandemic [35–40]: Low education and not speaking the language in the country of residence is found to be significantly associated with increased belief in COVID-19 misinformation. Beyond that, higher social disadvantage and lower digital health literacy are significantly linked to an agreement with misinformation [36]. Further, a cross-sectional study conducted in Bangladesh, found that people with low education, the elderly, people with chronic diseases, and people living in slums showed more vaccine hesitancy and a more negative attitude towards the COVID-19 vaccine than other population groups [37]. Moreover, COVID-19-specific risk perception was found to be higher in people with higher education and employed people in Sub-Saharan Africa and the Diaspora [38]. Similarly, COVID-19 risk perception was significantly higher in people with higher education and higher economic status among the Iranian population [40]. And to show another example, people older than 60 years were less likely to use trustworthy information sources than younger participants in a study conducted in the U.S. Moreover, low education and unemployment were significantly associated with a lower variety of information sources [39].

There are existing reviews, looking at communication inequalities in vulnerable groups during the COVID-19 pandemic [41], and single studies assessing communication inequalities linked with health disparities (CIHD) in vulnerable groups during the COVID-19 pandemic exist [42–44]. But to date, we are not aware of any review that summarizes not only communication inequalities in vulnerable groups but also their potential impact on health disparities, as it was done in past disease outbreaks [26, 31]. It is further unknown if there are gaps in research on specific vulnerable population groups or whether different vulnerable groups participated equally in studies assessing CIHD during the COVID-19 pandemic.

Within the overall objective of informing possible communication interventions for vulnerable groups and future research, this review aims to

- map and summarize existing evidence on the link between communication inequalities and health disparities in vulnerable groups during the COVID-19 pandemic and,
- identify research gaps that need to be addressed to establish conclusive knowledge on this link.

Specifically, the scoping review aims to answer the following research questions:

i) RQ1: To identify research gaps, what vulnerable groups, indicators of communication inequalities, and health outcomes are assessed in currently available research about the link between communication inequalities and health disparities in vulnerable groups during the COVID-19 pandemic?
ii) RQ2: Based on the results of current research about the link between communication inequalities and health disparities in vulnerable groups during the COVID-19 pandemic:
  o What vulnerable groups are affected by communication inequalities linked with health disparities?
  o What communication inequalities have been identified to affect vulnerable groups?
  o What health disparities are linked with identified communication inequalities in vulnerable groups?

## Methods

A scoping literature review was conducted to attain the aims outlined. This method is justified by the fact that after only two years of primary research during the COVID-19 pandemic, there is not sufficient evidence on the topic expected to allow a conclusive analysis. The scoping method addresses the objective of mapping and summarizing existing literature and identifying research gaps [45]. The guidelines of PRISMA extension for scoping reviews PRISMA ScR were followed in developing this study [45]. The protocol of this scoping review was not registered and is not accessible publicly.

### Conceptual framework and operationalization

**Fig. 1:**
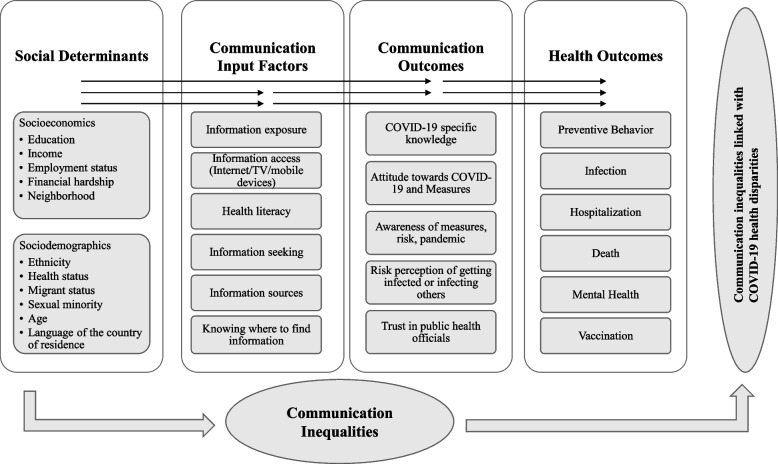
Hypothesized conceptual framework of communication inequalities linked with health disparities. The framework is derived from the Structural Influence Model of Viswanath et al. [23] and its adaptations by Lin et al. [26], Savoia et al. [31], and Kontos et al. [25]. Social determinants impact communication input factors, communication outcomes, and health outcomes, which can lead to communication inequalities and health disparities in vulnerable groups during the COVID-19 pandemic. The black arrows indicate the relationships investigated in this review as defined in the inclusion and exclusion criteria

The SIM by Viswanath et al. was adapted to serve as a conceptual framework for this scoping review [23]. Social determinants and health outcomes indicated in the conceptual framework (Fig. 1) are derived from literature identifying health disparities in socially vulnerable groups during the COVID-19 pandemic (Table 1). They are grouped into socioeconomic and sociodemographic factors, similar to the SIM [23]. Socioeconomic factors usually encompass education, household income/financial hardship, employment status, and neighborhood, whereas ethnicity, age, health status, migrant status, language, and sexual minority are considered sociodemographic factors [46, 47].

Following the recommendations from health communication evaluation literature [48], communication input factors and communication outcomes serve as indicators for communication inequalities in the conceptual framework. The individual items for the two indicators are derived from SIMs adapted for similar studies in other health crises [25, 26, 31] and were hypothesized for the COVID-19 pandemic with support from a senior researcher and health communication lecturer at the University of Lucerne. Social determinants are indicating vulnerable groups only when education, for example, is low but not, when it is high. The same accounts for communication input factors, communication outcomes, and health outcomes as they are indicating inequalities and disparities only in one direction. All hypothesized indicators for vulnerable groups, communication inequalities, and health disparities are derived from the conceptual framework and are displayed in Additional file 1. A manual literature search confirmed the link between the social determinants, communication input factors, and communication outcomes hypothesized for the conceptual framework [35–40, 49–51].

### Inclusion and exclusion criteria

Publications met inclusion criteria when they were empirical, conducted in the adult population (> age 18) in 2020 or later during the COVID-19 pandemic, and when they assessed either relationships

- between social determinants, communication input factors, and health outcomes,
- between social determinants, communication outcomes, and health outcomes or
- between social determinants, communication input factors, communication outcomes, and health outcomes, as indicated in Fig. 1.

Intervention studies to overcome communication inequalities were excluded as their identification is beyond the objectives of this review. Also, gray literature (e.g., governmental reports, newsletters, dissertations) and unpublished studies were excluded, such as studies not published in English.

### Search strategy

**Fig. 2:**
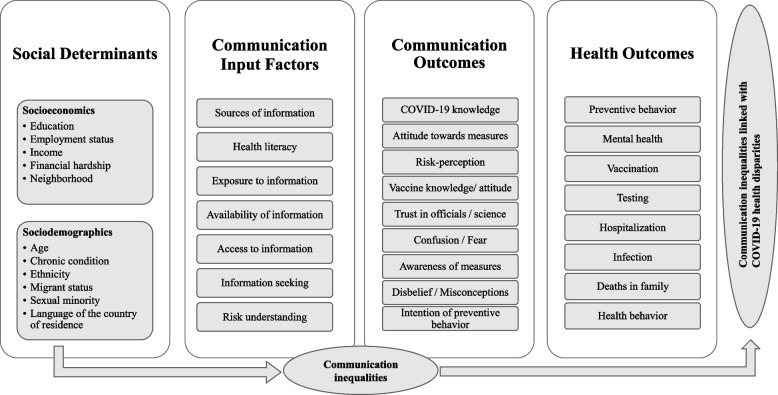
PRISMA Flow Diagram of search and screening results [54]

Three main search terms (*vulnerable groups, communication inequalities,* and *COVID-19*) were derived from the research questions, representing the population, context, and concepts for the scope of this review [45]. A search string was created for all three search terms using synonyms, subordinate terms, superordinate terms, other related keywords, and, truncation. The conceptual framework (Fig. 1) therefore served as a foundation. The three search strings were combined into one using the function "AND" and were adjusted for the bibliographic databases PubMed and PsycInfo with the support of a librarian from the University of Lucerne, e.g., by using Medical Subject Headings (MeSH) for PubMed. The two final search strings for each database are presented in Additional file 2. An external researcher from the University of Basel peer-reviewed the search strings following the Peer Review of Electronic Search Strategies (PRESS) checklist [52] before the search was executed by the first author of this review on November 18, 2021, on PubMed, and November 30, 2021, on PsycInfo. Electronic filters restricted the search to 2020 and 2021, the adult population (> age 18), full papers, and papers published in English. The search results were exported to a Zotero database, automatically identifying and removing duplicates. Eligible studies for the title/abstract screening were uploaded and screened by the first author in the web- and mobile application Rayyan [53]. To increase consistency and avoid bias, an external health science researcher from the University of Lucerne independently screened 10% of the literature before title/abstract screening. Conflicts were solved with discussions until there was agreement on all items. After title/abstract screening, additional papers were identified in reference lists of included studies and through hand search by the first author (between November 30, 2021, and February 2, 2022). Eligible studies were saved in a new Zotero database for full-text screening. A PRISMA flow diagram was used for documenting the search process (Fig. 2) [54].

### Data extraction

During the full-text screening, the first author extracted the data from included studies in a data-charting form. A critical appraisal of included studies was waived. The data charting form was developed for this review based on the conceptual framework (Fig. 1). It was discussed and updated continuously in regular meetings with an experienced researcher to ensure the extraction of all relevant variables for the analysis. In the final document, the population under examination, study design/methods, assessed social determinants, communication input factors, communication outcomes, the found relationships and health outcomes were extracted besides year, first author, and title.

### Data analysis

The extracted variables were grouped into social determinants, communication input factors, communication outcomes, and health outcomes predefined in the conceptual framework (Fig. 1). When deciding on the eligibility for a group, the operationalizations of found variables were considered. Since some of the variables did not fit into any of the existing groups in the conceptual framework but were still relevant for the review, some new groups were created. These include "availability of information" and "risk understanding" as communication input factors, "trust in science", "confusion/fear", "disbelief/misconceptions" and "intention of preventive behavior" as communication outcome factors, and "testing", "deaths in the family" and "health behavior" as health outcomes. The grouping of variables can be traced in Additional file 3. Observed associations between variables were classified into four main categories: Communication inequalities linked with health disparities (CIHD), Communication inequalities without a link to health disparities (CI), Health disparities without a link to communication inequalities (HD), and No communication inequalities or health disparities (NCIHD). In Table 2, the categories are described in more detail. CIHD, CI, and HD were structured based on social determinants and displayed in tables. NCIHD were excluded from further analysis. Proportions (pa) of found CIHD, CI, and HD were calculated based on the frequency they were assessed in identified studies (see Additional file 4).

## Results

### Included studies

The database search yielded 1490 studies. After removing 31 duplicates, 1459 papers were screened for title and abstract as described in the search strategy, of which 227 were included for full-text screening. Hand search and reference screening yielded 41 studies, of which 37 studies were additionally eligible for full-text screening. Of the total 264 studies screened for full-text, 92 papers were finally included for data extraction and analysis [43, 44, 55–144]. The search process is documented in the PRISMA flow diagram in Fig. 2. All included studies are listed in the data extraction form in Additional file 5.

### Characteristics of included studies

Among the 92 included studies, 42 were published in 2021 and 45 in 2020. All studies are observational and either designed cross-sectionally (*n* = 88) or as panel projects (*n* = 4). Most used quantitative methods (surveys) for data collection (*n* = 86), with 45 of them conducted online only. Six studies applied in-person qualitative methods (focus groups/interviews). The studies were conducted in 35 countries covering five continents, most of them stemming from Asia (*n* = 36), Africa (*n* = 29), and Northern America (*n* = 18). Fewer studies were conducted in Europe (*n* = 6) and Southern/Central America (*n* = 3). On a country level, most studies were conducted in the United States (U.S.) (*n* = 17) followed by China (*n* = 9), Saudi Arabia (*n* = 9), Ethiopia (*n* = 7), and India (*n* = 5). All countries can be viewed in the data-charting form in Additional file 5. Most studies were population-based (*n* = 60). Another group of studies researched specific vulnerable population groups (*n* = 26). Among them, some focused on people living with chronic conditions (HIV, diabetes, mental illness, cancer, disabilities, or diverse conditions) (*n* = 11), on adults > 60 years (*n* = 6), on ethnic minorities (Black, Latinx) (*n* = 5), migrant populations (refugees, migrants) (*n* = 2), people living in deprived neighborhoods (slums) (*n* = 1), and low-income populations (*n* = 1). A third group of studies recruited participants considered to be neutral regarding vulnerability to communication inequalities during the COVID-19 pandemic (e.g., outpatient hospital visitors) (*n* = 6).

### Descriptive depiction of extracted variables

**Fig. 3:**
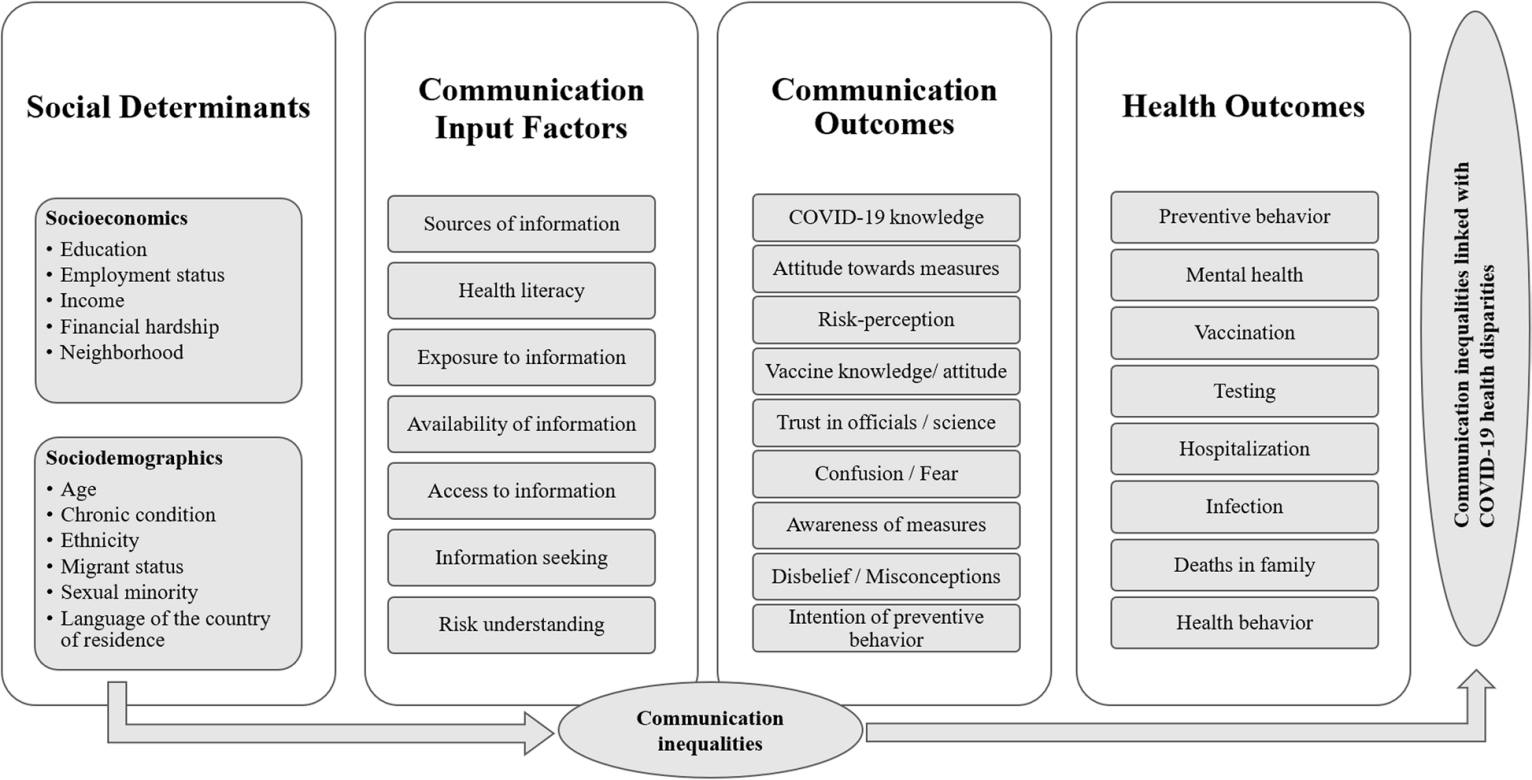
Framework of communication inequalities linked with health disparities, summarizing all variables identified in included studies. The framework is based on the conceptual framework developed for this study which is adapted from the SIM by Viswanath et al. [23] and its adaptations by Lin et al. [26], Savoia et al. [31], and Kontos et al. [25]

The following section addresses the first research question, presenting assessed variables in included studies. Figure 3 provides an overview of the grouped variables extracted, structured in the conceptual framework.

Most studies assessed more than one social determinant. Education was assessed most often (*n* = 82), followed by age (*n* = 64), employment status (*n* = 41), income (*n* = 36), chronic condition (*n* = 22), and ethnicity (*n* = 21). Migrant status (*n* = 6), financial hardship (*n* = 3), sexual minority (*n* = 2), language of the country of residence (*n* = 2), and neighborhood (*n* = 1) were less frequently assessed.

Regarding indicators for communication inequalities, most of the studies investigated communication outcomes only (*n* = 72). Some studies assessed both indicators (*n* = 17) or communication input factors only (*n* = 3). Among communication input factors, source of information was assessed most often (*n* = 7), before health literacy (*n* = 6), exposure to COVID-19 information (*n* = 5), availability of relevant/understandable information (*n* = 3), access to information (*n* = 2), information seeking (*n* = 2), and risk understanding (*n* = 1). The most frequently assessed communication outcomes were COVID-19 knowledge (*n* = 72) and attitude (*n* = 37). Risk perception was assessed in 14 studies. Other communication outcome variables that were found are vaccine knowledge/attitude *n* = 7), trust in officials/science (*n* = 5), confusion/fear (*n* = 4), awareness of measures (*n* = 4), disbelief/misconceptions (*n* = 4), and intention to preventive behavior (*n* = 1).

The health outcome assessed most frequently was preventive behavior (*n* = 84). Others are mental health (*n* = 5), vaccination (*n* = 4), testing (*n* = 2), hospitalization (*n* = 1), infection (n = 1), COVID-19 deaths in family (*n* = 1), and general health behavior (*n* = 1).

### Descriptive depiction of found relationships

This section is dedicated to answering the second research question, summarizing the results found in the included studies. Seventeen studies did not find any communication inequality or health disparity (all associations categorized as NCIHD) and therefore are excluded from further analysis. Three of them did not find significant results [142–144]. The remaining 14 studies found relationships not considered inequalities or disparities, as stated in Table 2 [128–141]. An example is a cross-sectional study in an HIV community in Kigali, Rwanda, assessing knowledge, attitude, and preventive behavior with face-to-face surveys [137]. The findings are high knowledge in 97%, a positive attitude in 74%, and good preventive practice in 90% of the participants. The positive communication outcomes and health outcomes in participants living with HIV do not indicate communication inequalities or health disparities based on the social determinant of living with a chronic condition.

**Table 2:** Categorization of findings from included studies

	**Quantitative population-based studies**	**Quantitative studies in vulnerable populations**	**Qualitative interviews / focus groups in vulnerable populations**
CIHD	Statistically significant association of a social determinant with • a negative communication input factor, negative communication outcome, or both, • and a negative health outcome	> 50% of the participants reporting • a negative communication input factor, negative communication outcome, or both, • and a negative health outcome	Participants mentioning • a negative communication input factor, negative communication outcome, or both, • and a negative health outcome
CI	Statistically significant association of a social determinant with • a negative communication input factor, negative communication outcome, or both, without significant association with a negative health outcome	> 50% of the participants reporting • a negative communication input factor, negative communication outcome, or both, without a negative health outcome	Participants mentioning • a negative communication input factor, negative communication outcome, or both, without a negative health outcome
HD	Statistically significant association of a social determinant with • a negative health outcome without significant association with a negative communication input factor or negative communication outcome	> 50% of the participants reporting • a negative health outcome without a negative communication input factor or negative communication outcome	Participants mentioning • a negative health outcome without a negative communication input factor or negative communication outcome
NCIHD	Statistically significant association of a social determinant with • a positive communication input factor, positive communication outcome, • a positive health outcome, • or both Or No significant association of any social determinant with any communication input factor, communication outcome, or health outcome	> 50% of the participants reporting • a positive communication input factor, positive communication outcome, • a positive health outcome, • or both Or Participants did not mention any communication input factor, communication outcome, or health outcome	Participants mentioning • a positive communication input factor, positive communication outcome, • a positive health outcome, • or both Or Participants did not mention any communication input factor, communication outcome, or health outcome

#### Communication inequalities linked with health disparities (CIHD)

The largest group of studies found a link between communication inequalities and health disparities in vulnerable groups (*n* = 45). Table 3 shows all CIHD found in vulnerable groups.

**Table 3:** Communication inequalities linked with health disparities (CIHD) in vulnerable groups

Social determinants	Communication input factors	Communication outcomes	Health outcomes	References
**Low education**		Low knowledge	Low preventive behavior	[55, 56, 64, 68, 69, 76–78, 80, 86–90, 92, 95–97]
		Low knowledge and negative attitude	Low preventive behavior	[57, 74, 82]
		Negative attitude	Low preventive behavior	[44, 58, 83]
		Disbelief	Low preventive behavior	[60]
	Low risk-understanding		Low preventive behavior	[62]
	Low e-health literacy, Low information seeking		Low preventive behavior	[93]
	Lack of relevant information	Distrust, low knowledge, negative attitude	Low vaccination rate	[66]
		Distrust in public health officials, distrust in vaccine	Low vaccination rate	[65]
**Low income**		Low knowledge	Low preventive behavior	[75, 85, 88, 92]
		Distrust in public health officials, distrust in vaccine	Low vaccination rate	[65]
	Lack of relevant information	Distrust, low knowledge, negative attitude	Low vaccination rate	[66]
		Disbelief	Low preventive behavior	[60]
		Negative attitude	Low preventive behavior	[83]
	Low risk-perception		Low preventive behavior	[91]
	Low e-health literacy, Low information seeking		Low preventive behavior	[93]
		Low knowledge, negative attitude	Low preventive behavior	[98]
**Ethnic minority**		Low vaccination, high skepticism, distrust in government	Low testing rate	[61]
	Lack of relevant information availability	Distrust, low knowledge, negative attitude	Low vaccination rate	[66]
	Lack of relevant information availability	Mistrust, low knowledge	Low vaccination rate	[67]
		Low knowledge	High infection, low preventive behavior	[70]
	Untrustworthy information source	Disbelief, misinformation	Hospitalization	[72]
		Confusions	Mental health problems	[94]
		Distrust in public health officials, distrust in vaccine	Low vaccination	[65]
**Chronic condition**		Trust in official information sources	Low preventive behavior	[81]
	Confusion		Mental health problems	[43]
		Low health literacy	Low preventive behavior	[73]
**Higher age (> 60 years)**		Low knowledge	Low preventive behavior	[71, 89]
**Unemployed**		Low knowledge	Low preventive behavior	[59]
		Negative attitude	Low preventive behavior	[80]
**Sexual minority**		Low risk-awareness	Low preventive behavior	[63]
**Financial hardship**		Distrust in public health officials, distrust in vaccine	Low vaccination rate	[65]
**Deprived neighborhood**		Low knowledge	Low preventive behavior	[84]

The vulnerable group identified most often (in 29 studies) to be affected by CIHD is people with low education. Of the studies that assessed education as a social determinant, (pa 35%) found CIHD in this vulnerable group. Among them, 21 studies identified people with lower education as having lower knowledge and less preventive behavior than those with higher education, which is the most prevalent CIHD. One example is a cross-sectional study assessing knowledge and preventive behavior toward COVID-19 among pregnant women in Ghana [78]. Pregnant women living with a chronic disease presented more preventive behaviors than healthy women, whereas women with low education had significantly lower knowledge and applied less preventive behavior than other participants.

Eleven studies found CIHD in persons with low income (pa 31%). The link found most often in this group was low income associated with insufficient knowledge and low preventive behavior. An example is a study by Guo et al., assessing socioeconomic differences in e-health literacy and COVID-19 preventive behavior in a cross-sectional survey study in Hong Kong [93]. Low socioeconomic status was significantly associated with diminished e-health literacy, reduced information seeking, and lower preventive behavior than participants with high socioeconomic status.

Seven studies identified CIHD in ethnic minorities (pa 33%). One example is the qualitative study by Cervantes et al., assessing the experiences of Latinx individuals hospitalized for COVID-19 in telephone interviews [72]. Participants named misinformation and disbelief due to social media consumption as causes of getting infected and hospitalized. Other reasons mentioned were the desire to comply with social and cultural norms, high-density housing, and the fear of financial constraints when not working due to the risk of infection.

Three studies found CIHD in people living with a chronic condition (pa 13%). A study conducted in the U.S. assessed the impact of the COVID-19 pandemic on men diagnosed with HIV [43]. In qualitative interviews, this population of men with HIV reported confusion about COVID-19 information and increased mental health problems since the pandemic started. However, participants showed high knowledge and risk perception, used trustworthy information sources, and showed high preventive behavior.

CIHD in people of age higher than 60 years were found in two studies (pa 3%). A population-based cross-sectional study conducted in Iran is one example [89]. It assessed knowledge, attitude, risk perception, and preventive COVID-19 practices in face-to-face surveys. The findings were a lower level of knowledge and preventive practices in retired people and people with lower education.

Two studies identified CIHD in unemployed people (5%). One of them investigated COVID-19 knowledge, attitude, and practice in the Egyptian population in a cross-sectional study using online and face-to-face surveys [80]. Low income was associated with significantly lower attitude, unemployment with both, significantly lower attitude, and lower preventive practice than employed participants. The same study found decreased knowledge and practice in people with low education. In sexual minorities, people living with financial hardship, and people living in deprived neighborhoods, CIHD were found in one study each.

#### Communication inequalities without a link to health disparities (CI)

All CI are summarized in Table 4, with insufficient knowledge in low educated people being the most prevalent CI. Twenty-five studies found CI but no CIHD, and eleven studies found CI for some social determinants among CIHD for other social determinants.

**Table 4:** Communication inequalities without a link to health disparities (CI) based on vulnerable groups

Social determinants	Communication input factors	Communication outcomes	References
**Low education**		Low knowledge	[75, 100, 104, 107, 109, 112–115, 118, 122]
		Low knowledge and negative attitude	[99, 110, 116]
		Negative attitude	[88, 98]
		Low risk-awareness	[111]
	Low exposure to information	Misinformation	[117]
**Low income**		Low knowledge	[55, 70, 115, 116, 120, 122]
	Low exposure to information	Misinformation	[117]
		Negative attitude	[80]
**High age**		Low knowledge	[114, 122]
		Low knowledge and negative attitude	[113]
		Vaccine hesitancy	[106]
		Negative attitude	[104]
		Low risk-perception	[102]
	Low e-health literacy, Low information seeking		[93]
**Ethnic minority**		Low knowledge and negative attitude	[116]
		Low risk-awareness	[108]
		Low knowledge	[55]
**Unemployed**		Low knowledge	[77, 90, 92, 121]
		Low knowledge and negative attitude	[119]
		Negative attitude	[83]
**Chronic condition**		Low knowledge	[79, 105]
		Low awareness, low knowledge	[103]
	Less information seeking		[93]
**Migrant status**	Low health literacy		[101]

For example, Bazaid et al., who assessed COVID-19 knowledge and practice in the Saudi Arabian population, found CI based on low education with a significant association of low education with insufficient knowledge [75]. Low education was not related to decreased practice. The same study found CIHD based on low income with significantly lower knowledge and preventive behavior in people with low income than those with high income.

#### Health disparities without a link to communication inequalities (HD)

Although all included studies assessed indicators for communication inequalities, some found health disparities that are not linked with communication inequalities. Five studies found HD only. Seven studies found HD for some variables among CI or CIHD for others. All HD found are displayed in Table 5.

**Table 5:** Health disparities without a link to communication inequalities based on vulnerable groups

Social determinants	Health outcome	References
High age	Low preventive behavior	[87, 91, 120, 123, 125, 126]
Low education	Low preventive behavior	[124, 127]
Chronic condition	Low preventive behavior	[62, 126]
Migrant status	Low preventive behavior	[58, 91]
Low income	Low preventive behavior	[80]
Unemployed	Low preventive behavior	[91]
Ethnic minority	Low preventive behavior	[117]

An example is a cross-sectional study conducted in the U.S. assessing health literacy, knowledge, and preventive behaviors in adults with chronic conditions using telephone surveys [126]. Results are good COVID-19 knowledge in 71% of the participants, whereas only 38% reported applying preventive behaviors. Having more than one chronic condition and being Black or Latinx was associated with yet less preventive behavior.

## Discussion

The COVID-19 pandemic has aggravated existing health disparities as elaborated in Table 1 (e.g. increased mortality [9], hospitalizations [10], infection rates [7], and lower preventive behavior [14] in socially vulnerable population groups [7–22]). Communication inequalities are likely to play a vital role as underlying mechanisms of this relationship [35]. This scoping review identifies and maps current literature assessing the link between communication inequalities and health disparities in vulnerable groups during the COVID-19 pandemic and summarizes the results of identified studies. Ninety-two studies assessing this link were found.

The first finding is that most of the included studies are conducted in the U.S., China, Saudi Arabia, Ethiopia, and India. Second, it was found that current research focuses on identifying CIHD in people with low education and on assessing communication outcomes rather than communication input factors, with knowledge being the most prevalent communication outcome. Moreover, current research primarily assesses preventive behavior as a health outcome possibly linked with communication inequalities in vulnerable groups. The existing MeSH term "Health knowledge, attitudes, practice" (KAP) used for this review might explain the high prevalence of these variables [145]. Another reason for the high proportion of KAP studies might be the WHO's guide to developing questionnaires to systematize the collection and use of KAP data [146].

Third, around half of the analyzed studies found CIHD in vulnerable groups during the COVID-19 pandemic. The other half of the included studies found part of the link hypothesized in the conceptual framework (Fig. 1). The second-largest group of studies found CI only, and others found only HD or none. This result indicates a critical role of communication as mediating factor between social determinants of health and health disparities on the one hand. It provides some scientific support for the conceptual framework developed for this review and the SIM by Viswanath et al. [23]. On the other hand, the identified CI show that unequal communication does not necessarily pay out as adverse health outcomes, or at least not as the ones assessed in identified studies. Found HD reveal that well-informed people still experience other barriers to health, such as the (e.g., financial) capacity to act upon preventive measures. This effect must not be neglected [147], especially if considering the high proportion of studies conducted in rather low-income countries. Another aspect found in this group of studies is that people older than 60 years are most affected by HD. Belonging to the medical risk group for COVID-19, they were targeted explicitly in communication strategies which can explain the missing link with communication inequalities [148, 149]. The HD experienced by older adults might be explained by reduced health-care seeking due to fear of infection or stigmatization related to discriminatory risk communication in the context of the COVID-19 pandemic [149].

Fourth, people with limited education are the most affected vulnerable group of CIHD and CI. The most observed communication inequality, with or without a link to a health disparity, is decreased knowledge in people with low education. The most prevalent health disparity linked with communication inequalities is low preventive behavior. Therefore, it is not surprising that the full link of CIHD found most often is low education associated with deficient COVID-19 knowledge and low preventive behavior. These results are similar to those found in a review on the H1N1 pandemic, where low education was linked with insufficient knowledge and low preventive behavior [26]. Other social determinants, such as ethnicity and income related to CI and HD, were identified in this review, too [26]. The findings further confirm the scientifically well-supported knowledge gap hypothesis that increased information flow increases knowledge in highly educated populations, exacerbating societal knowledge differences [150, 151]. Although similar results were found in the H1N1 pandemic, the COVID-19 pandemic is particularly prone to communication inequalities due to two challenges: Firstly, the public had to interpret and process raw scientific information conveyed shortly after its discovery [35]. Secondly, social media sped up the dissemination of information, disinformation, and misinformation [35]. Therefore, it is likely that closing identified research gaps will reveal more CIHD and CI than the ones found in the currently available literature.

The main research gaps identified in this scoping review are a lack of studies conducted in European, as well as Central and Southern American countries. Further, few studies assessed migrant status, financial hardship, speaking the language in the country of residence, sexual minorities, and living in deprived neighborhoods. This result might indicate that some of these population groups are hard to involve, so their participation could be waived due to scarce resources [152]. However, their involvement is vital to identifying inequalities and disparities. Another gap identified is the lack of studies assessing communication input factors. One explanation might be the questionable reliability of self-reported input factors, such as health literacy, exposure, or access to information [48]. Therefore, researchers might prefer assessing communication outcomes as indicators for communication inequalities as this may be more straightforward. But, considering the definition of communication inequalities used for this review, communication input factors can be seen as more direct indicators of communication inequalities [26]. Their assessment is essential to understanding the underlying mechanisms of emerging inequalities and disparities in vulnerable groups. Some interventions to overcome identified communication inequalities and health disparities in vulnerable groups during the COVID-19 pandemic have been developed [153–156]. However, closing these research gaps timely is vital to analyze the information environment in real-time and develop specifically targeted intervention strategies to prevent CIHD [31].

This scoping review comes with some limitations. Because of limited time and financial resources, the search was restricted to two electronic databases. Moreover, the search strings were limited to title and abstract to achieve feasible search results for the scope of this study. Relevant studies might have been missed when they only mentioned keywords in the main text. Also, studies published in languages other than English or in December 2019 could have been missed due to the restricted search string. Hand search and reference screening of the most relevant studies were performed to compensate for these limitations. Full-text screening, data extraction, and categorization were done by only one person, increasing the risk of errors and biases. In order to address this and to avoid a limited validity of the results, decisions were regularly discussed with experienced senior researchers and professors, including the Co-Authors. Another limitation is the exclusion of gender and sex from the social determinants of CIHD. This is justified as it might depend on the socio-cultural context women live in if they are at increased risk of CIHD. The evaluation of each study in this regard is beyond the scope of this review. However, their exclusion risks missing communication inequalities in a possibly vulnerable population.

A further limitation is that the scientific relevance of the summarized results of the studies included is limited for several reasons. First, they cannot be interpreted as conclusive as a quality assessment of included studies was waived. Second, all analyzed studies are observational, and thus, observed relationships cannot be interpreted as causal. Third, most of the studies are population-based, using online surveys. Population-based studies may show statistically significant differences between vulnerable and other population groups and thus point out inequalities more clearly than studies with vulnerable participants only. Nevertheless, vulnerable population groups are often hard to reach, especially in population-based online surveys [152]. Therefore, the results of these studies might be biased due to the underrepresentation of vulnerable participants. Fourth, the variables in the CIHD found most often are the most frequently assessed ones at the same time. Other links might be as prevalent but are not investigated in studies yet. Fifth, different measurements were used between the studies to assess variables, and thus, what is defined as "low," "insufficient," or "deficient" may vary too. Therefore, the comparability of results between the studies is reduced. And sixth, the categorization and threshold of what findings are considered inequalities or disparities are defined for this review only, based on the decision of one person. However, the categorization was done systematically and is documented comprehensibly.

The conceptual framework (Fig. 1) was not tested in previous projects. But, it is based on the scientifically supported SIM, other similar frameworks, and recent COVID-19 evidence and is now at least partly supported by the results of this study [23, 26, 31]. Therefore, it is considered a strength of this review. It can be used for future research projects to identify CIHD in public health emergencies. Another strength is that this study covers a highly relevant problem. The provided literature summary identifies research gaps in a time point where they can still be minimized in real-time. The results found in studies highlight the importance of the topic and the relevance of investing more scientific resources in identifying communication inequalities linked with health disparities in vulnerable groups – now and in future public health emergencies.

The fact that this study could show that communication can actually have an impact on health outcomes, is considered a major strength. Although research gaps in vulnerable groups that have to be closed were identified, it can be concluded, that vulnerable population groups are especially at risk of adverse health outcomes. Therefore, it is crucial for health institutions to invest in effective communication and to find ways to reach all population groups through targeted interventions. One way that researchers and public health institutions can get engaged is by investing in community involvement. Through community involvement, vulnerable groups can be better reached to participate in studies. At the same time, actively involving vulnerable groups in the planning, implementation, and evaluation of measures to prevent and combat CI and CIHD can enable effective communication during a public health crisis such as the COVID-19 pandemic [157].

## Conclusions

Current literature about CIHD in vulnerable groups during the COVID-19 pandemic focuses on assessing CIHD associated with low education. Communication outcomes are primarily assessed as indicators of communication inequalities. Half of the identified studies found CIHD in vulnerable groups. Other studies found CI, HD, or none. Despite some limitations of this review, these results show that effective communication is not only an ideal but can indeed have a detrimental effect on health outcomes.

More studies assessing CIHD are needed from European and Central and Southern American countries as well as on people with migrant status, financial hardship, not speaking the language in the country of residence, sexual minorities, and living in deprived neighborhoods. Future studies should include assessing communication input factors to identify more direct intervention targets. Closing these research gaps is essential for preventing CIHD in vulnerable groups. Based on the currently available evidence, people with low education are most affected by CIHD and CI during the COVID-19 pandemic. However, this study could show that several other vulnerable population groups are at increased risk of communication inequalities and health disparities during a public health crisis. It is crucial for researchers to include also vulnerable groups in future studies and for public health institutions to identify means to reach all population groups with targeted, effective interventions to diminish communication inequalities and health disparities in public health crises.

## Supplementary Information

**Additional file 1.** Indicators.

**Additional file 2.** Search strings.

**Additional file 3.** Categorization.

**Additional file 4.** Proportions.

**Additional file 5.** Data-charting form.

## Data Availability

All data collected and analyzed for this review are included in this published article and its supplementary information files (Additional files 1–5).

## Abbreviations

CI: Communication inequalities without a link to health disparities
CIHD: Communication inequalities linked with health disparities
HD: Health disparities without a link to communication inequalities
KAP: Health knowledge, attitudes, practice
MeSH: Medical Subject Headings
n: Number
NCIHD: No identified communication inequalities or health disparities
pa: Proportion of found relationships in studies assessing them (n relationships found/n relationships assessed)
SIM: Structural Influence Model
U.S.: United States of America
WHO: World Health Organization
