# Supplementary material for: Communication inequalities and health disparities among vulnerable groups during the COVID-19 pandemic - a scoping review of qualitative and quantitative evidence
Source: BMC Public Health. 2023 Mar 6;23:428. doi: 10.1186/s12889-023-15295-6 (PMC9986675; doi:10.1186/s12889-023-15295-6)
Supplement: Supplementary file 1 — Additional file 1. Indicators. [file 12889_2023_15295_MOESM1_ESM.docx]

# Additional file 1: Indicators

**List of defined indicators for vulnerable groups, communication inequalities, and health disparities based on the conceptual framework. This table does not display any associations among the indicators.**

| **Vulnerable groups** | | **Communication inequalities** | | **Health disparities** |
| --- | --- | --- | --- | --- |
| **Socioeconomic vulnerabilities** | **Sociodemographic vulnerabilities** | **Negative communication input factors** | **Negative communication outcome factors** | **Negative health outcome** |
| Low education | Belonging to an ethnic minority in the country of residence | Low exposure to information about COVID-19 | Reduced knowledge and misconceptions about COVID-19, COVID-19 preventive measures, groups with medical risks from infection, transmission, and origin of the virus | Not/less applying measures to prevent an infection with COVID-19 |
| Low income | Living with a chronic condition | Low access to information about COVID-19 due to lack of access to internet, TV, and other mobile devices | Having a negative attitude towards COVID-19 preventive measures, governmental and political measures | Being infected with COVID-19 |
| Being unemployed | Having migrant status in the country of residence | Having a low health literacy (knowledge, motivation, and competence to access, understand, elaborate, and use health information to make health-related decisions in daily life (1)) | Being aware of preventive measures, the existing pandemic | Being hospitalized with COVID-19 |
| Living with financial hardship | Belonging to a sexual minority regarding gender and sexual orientation | Reduced information-seeking behavior | Perceived susceptibility, perceived severity of getting infected or of infecting others with COVID-19 | Dying from COVID-19 |
| Living in a deprived neighborhood | Being older than 60 years of age | Relying on unofficial information sources | Having trust in public health officials | Experiencing worse mental health related to COVID-19 (e.g. Anxiety, depression, PTSD…) |
|  | Not having profound knowledge of the language in the country of residence | Not knowing where to find information about COVID-19 |  | Getting the COVID-19 vaccine |
