# Supplementary material for: Communication inequalities and health disparities among vulnerable groups during the COVID-19 pandemic - a scoping review of qualitative and quantitative evidence
Source: BMC Public Health. 2023 Mar 6;23:428. doi: 10.1186/s12889-023-15295-6 (PMC9986675; doi:10.1186/s12889-023-15295-6)
Supplement: Supplementary file 2 — Additional file 2. Search strings. [file 12889_2023_15295_MOESM2_ESM.docx]

# Additional file 2: Search strings

| **PubMed**  Executed on November 18, 2021 | **PsycInfo**  Executed on November 30, 2021 |
| --- | --- |
| (("inequalit*"[Title/Abstract] OR "disparit*"[Title/Abstract] OR "inequit*"[Title/Abstract] OR "equit*"[Title/Abstract] OR "Ethnic Groups"[MeSH Terms] OR "Chronic Disease"[MeSH Terms] OR "Socioeconomic Factors"[MeSH Terms] OR "migration*"[Title/Abstract] OR "Minority Groups"[MeSH Terms] OR "Sexual and Gender Minorities"[MeSH Terms] OR "gay"[Title/Abstract] OR "racial"[Title/Abstract] OR "elderly"[Title/Abstract] OR "aged"[Title/Abstract]) AND ("health knowledge, attitudes, practice"[MeSH Terms] OR "Access to Information"[MeSH Terms] OR "Social Media"[MeSH Terms] OR "Mass Media"[MeSH Terms] OR "Internet Access"[MeSH Terms] OR "Health Communication"[MeSH Terms] OR "Communication Barriers"[MeSH Terms] OR "media"[Title/Abstract] OR "knowledge"[Title/Abstract] OR "information"[Title/Abstract] OR "awareness"[Title/Abstract] OR "information seeking"[Title/Abstract] OR "health literacy"[MeSH Terms] OR "attitude*"[Title/Abstract] OR "risk perception"[Title/Abstract] OR "trust"[Title/Abstract] OR "information exposure"[Title/Abstract]) AND ("covid 19"[MeSH Terms] OR "covid19*"[Title/Abstract] OR "covid*"[Title/Abstract] OR "coronavirus"[Title/Abstract] OR "corona virus"[Title/Abstract] OR "coronavirus"[MeSH Terms])) AND ((fft[Filter]) AND (english[Filter]) AND (alladult[Filter]) AND (2020:2021[pdat])) | ((MAINSUBJECT.EXACT("COVID-19") OR MAINSUBJECT.EXACT("Coronavirus") OR ab(covid19) OR ab(coronavirus) OR ab(covid*)) AND (MAINSUBJECT.EXACT("News media") OR MAINSUBJECT.EXACT("Trust") OR ab("risk perception") OR MAINSUBJECT.EXACT("Access to information") OR MAINSUBJECT.EXACT("Consumer health information") OR MAINSUBJECT.EXACT("Health literacy") OR ab("health communication") OR ab(knowledge) OR ab(attitude) OR ab(awareness) OR ab(information) OR ab(media) OR ab("internet access") OR ab("communication barriers")) AND (MAINSUBJECT.EXACT("Chronic illnesses") OR MAINSUBJECT.EXACT("Socioeconomic factors") OR ab(inequit*) OR MAINSUBJECT.EXACT("LGBTQ studies") OR MAINSUBJECT.EXACT("Older people") OR MAINSUBJECT.EXACT("Minority & ethnic groups") OR ab(disparit*) OR ab(inequal*))) AND (stype.exact("Scholarly Journals") AND la.exact("ENG") AND su.exact("Adulthood (18 yrs & older)")) |
