# Supplementary material for: Communication inequalities and health disparities among vulnerable groups during the COVID-19 pandemic - a scoping review of qualitative and quantitative evidence
Source: BMC Public Health. 2023 Mar 6;23:428. doi: 10.1186/s12889-023-15295-6 (PMC9986675; doi:10.1186/s12889-023-15295-6)
Supplement: Supplementary file 3 — Additional file 3. Categorization. [file 12889_2023_15295_MOESM3_ESM.docx]

# Additional file 3: Categorization

**This table displays how the variables identified in the literature were grouped and categorized for the data analysis.**

| **Categories** | **Variables found in the literature** |
| --- | --- |
| **Social determinants** | |
| Ethnic minority | race, Black, Latinx, Hispanic, people of color |
| Chronic condition | disability, diabetes, HIV, chronic disease, cancer, severe mental illness, hypertension |
| Sexual minority | sexual minorities, men who have sex with men, bisexual, gay |
| Migrant status | nationality, refugee, migrants |
| Language of the country of residence | limited English proficiency (in the U.S.) |
| Income | monthly income quintiles, family income, income |
| Education | being able to read and write, education level, basic literacy, school degree, academic achievements |
| Financial hardship | poverty level, perceived SES, financial hardship |
| High age | 60 years and older |
| Neighborhood | Living in slums |
| Employment status | occupation (when being unemployed was provided as an option), employment status |
| **Communication input factors** | |
| Health literacy | critical health literacy, e-health literacy, health literacy |
| Availability of information | availability of relevant information about the own population for decision making regarding the vaccine, understanding information (availability of information in mother language), lack of information |
| Information seeking | seeking web-based information, seeking behavior |
| Exposure to information | exposure to risk information, exposure to misinformation, exposure to information |
| Access to information | access to information |
| Risk understanding | risk understanding |
| Sources of information | sources of information, social media as the main information source |
| **Communication outcome** | |
| COVID-19 knowledge | knowledge, misinformation, quarantine understanding, belief in misinformation |
| Trust in officials/science | trust in official COVID-19 information sources, distrust in government, distrust in science |
| Attitude towards measures | attitude towards COVID-19 measures |
| Awareness of measures | awareness of measures |
| Risk-perception | awareness of risks, perceived severity, perceived susceptibility |
| Intention of preventive behavior | intention of applying preventive measures |
| Disbelief / misconceptions | disbelief, misconceptions, (quarantine) understanding |
| Confusion / Fear | confusion about received COVID-19 information, fear of getting COVID-19, fear of COVID-19 |
| Vaccine knowledge/attitude | knowledge about vaccine, attitude towards the vaccine, vaccine hesitancy, vaccine intention, vaccine skepticism, distrust in vaccine |
| **Health outcomes** | |
| Preventive behavior | mask-wearing, hand washing, social distancing, reducing physical contacts, hand sanitizing, preventive practice, staying at-home measures, in-home precautions, self-isolation |
| Vaccination | taking the vaccine/Not taking the vaccine |
| Mental health | worsening previously existing mental health disorders |
| Infection | being or having been infected with COVID-19 |
| Hospitalization | being hospitalized with COVID-19 |
| Testing | getting tested for COVID-19 |
| Health behavior | alcohol and tobacco consumption |
| Deaths in family | number of deaths due to COVID-19 in family |
