# Supplementary material for: Communication inequalities and health disparities among vulnerable groups during the COVID-19 pandemic - a scoping review of qualitative and quantitative evidence
Source: BMC Public Health. 2023 Mar 6;23:428. doi: 10.1186/s12889-023-15295-6 (PMC9986675; doi:10.1186/s12889-023-15295-6)
Supplement: Supplementary file 4 — Additional file 4. Proportions. [file 12889_2023_15295_MOESM4_ESM.docx]

# Additional file 4: Proportions

**Proportions of CIHD, CI and HD based on social determinants**

| *Assessed social determinant* | *Frequency of assessments in studies* | *Frequency of found CIHD** | *Pa**** CIHD* | *Frequency of found CI*** | *Pa**** CI* | *Frequency of found HD**** | *Pa**** HD* |
| --- | --- | --- | --- | --- | --- | --- | --- |
| Education | 82 | 29 | 35.37% | 18 | 21.95% | 2 | 2.44% |
| Age | 64 | 2 | 3.23% | 7 | 10.94% | 6 | 9.38% |
| Employment | 41 | 2 | 4.88% | 6 | 14.63% | 1 | 0.02% |
| Income | 36 | 11 | 30.55% | 8 | 22.22% | 1 | 2.78% |
| Chronic disease | 22 | 3 | 13.36% | 4 | 18.18% | 2 | 9.09% |
| Ethnicity | 21 | 7 | 33.33% | 3 | 14.29% | 1 | 4.76% |
| Migrant population | 6 | 0 | 0% | 1 | 16.66% | 2 | 33.33% |
| Financial hardship | 3 | 1 | 33.33% | 0 | 0% | 0 | 0% |
| Sexual minorities | 2 | 1 | 50% | 0 | 0% | 0 | 0% |
| Language in the country of residence | 2 | 0 | 0% | 0 | 0% | 0 | 0% |
| Deprived neighborhood | 1 | 1 | 100% | 0 | 0% | 0 | 0% |
| **Communication inequalities linked with health disparities*  ***Communication inequalities without a link to health disparities*  ****Health disparities without a link to communication inequalities*  ***** Proportion of found relationships in studies assessing them (n relationships found/n relationships assessed)* | | | | | | | |
