# Supplementary material for: Communication inequalities and health disparities among vulnerable groups during the COVID-19 pandemic - a scoping review of qualitative and quantitative evidence
Source: BMC Public Health. 2023 Mar 6;23:428. doi: 10.1186/s12889-023-15295-6 (PMC9986675; doi:10.1186/s12889-023-15295-6)
Supplement: Supplementary file 5 — Additional file 5. Data-charting form. [file 12889_2023_15295_MOESM5_ESM.docx]

# Additional file 5: Data-charting form

| **Reference** | **1st author** | **Study design/ method** | **Population/**  **Country under study** | **Assessed social determinants** | **Assessed communi-cation input factors** | **Assessed communi-cation outcomes** | **Communication inequalities without a link to health disparities** | **Communication inequalities linked with health disparities** | **Health disparities without a link to communication inequalities** | **No communication inequalities or health disparities found** | **Assessed health outcomes** |
| --- | --- | --- | --- | --- | --- | --- | --- | --- | --- | --- | --- |
| (1) | Rhodes, Scott D. | cross-sectional, semi-structured interviews | gay, bisexual, and other men who have sex with men living with HIV in the U.S. | ethnicity, age, sexual orientation | information sources | knowledge, risk perceptions, confusion about information | x | People **living with HIV** are confused about information, Mental health issues related to COVID-19 were observed. | x | People living with HIV have high knowledge and risk perception and use trusted information sources. Preventive behavior was good. | preventive behavior and mental health |
| (2) | Okello, Gerald | cross-sectional, online survey | Ugandan population | education, age, employment | x | knowledge, attitude towards preventive measures | x | People with **low education** have a significantly more negative attitude towards preventive behaviors and are significantly less adherent to preventive behaviors than high educated people. | x | x | preventive behavior |
| (3) | Clements, John M. | cross-sectional, online survey | U.S. population | income, education, ethnicity, age | x | knowledge | **Being Black** and having **a lower incom**e is significantly associated with lower knowledge. | **Low education** is significantly associated with low knowledge and less mask-wearing. | x | Black people wear more masks than whites, although they have lower knowledge. | preventive behavior |
| (4) | Xu, Na | cross-sectional, online survey | Shaanxi province, China | education | x | knowledge | x | Low education is significantly associated with lower knowledge and less preventive behavior. | x | x | preventive behavior |
| (5) | Fatmi, Zafar | cross-sectional online survey | Pakistani population | age education (no literacy, no education), employment | x | knowledge, attitude | x | Not having **basic literacy** (some education) is significantly associated with lower knowledge and a more negative attitude towards preventive measures, as well as with less preventive measures. | x | x | preventive measures |
| (6) | Alnasser, Ali Hassan A. | cross-sectional web survey | Saudi Arabian population | education, age, nationality | x | knowledge, attitude | x | **Low education** is significantly associated with more negative attitudes and less preventive behavior. | **Non-Saudis** have lower practice than Saudis. | x | preventive measures |
| (7) | Alsaif, Bandar | cross sectional, online survey | Saudi Arabian population | chronic disease, occupation | x | knowledge, attitude | x | **Unemployed** people show significantly less knowledge and less preventive behavior than employed. Less knowledge - more general anxiety disorders. | x | x | preventive behaviors, mental health disorders |
| (8) | Al Zabadi, Hamzeh | cross sectional - social media survey | Palestinian population | education, income | x | quarantine understand-ding | x | **Low education** is significantly associated with poorer understanding of quarantine (disbelief) and lower adherence to staying at home. L**ow income** is significantly associated with less understanding and fewer in-home measures. | x | x | staying at home measures, in home precautions |
| (9) | Jimenez, Manuel E. | cross-sectional qualitative personal and group interviews | Black and Latinx in New Jersey, U.S. | Black and Latinx, income, education, age | information-seeking behavior | vaccination skepticism, distrust, awareness, knowledge | x | **POC** describe testing barriers due to not having a social security number, language, and other logistic barriers. Vaccination skepticism and distrust in government are very high. | x | x | testing and preventive behaviors, deaths in families |
| (10) | Rahman, Farah Naz | cross-sectional survey | Bangladeshis' population | education, disability | understanding risk communication | knowledge about preventive behavior | x | **Low-educated people** face significantly more problems with understanding risk communication and applying health-protective measures than higher educated. | **People with disability** show more problems in preventive behavior than people without disabilities. | x | preventive behavior |
| (11) | Ko, Nai-Ying | cross-sectional online survey | heterosexuals and sexual minorities in Taiwan | sexual minorities | x | awareness, attitude | x | **Sexual minorities** have significantly lower perceived susceptibility, fewer worries about covid 19, and apply fewer preventive measures | x | x | preventive behaviors |
| (12) | Sengeh, Paul | cross-sectional, survey | Sierra Leonean population | education, age | information sources, | knowledge | x | **Low educated** people have significantly less knowledge and apply fewer preventive behaviors than high educated. | x | x | preventive behavior |
| (13) | Nguyen, Kimberly H. | panel, household survey | U.S. population | income, financial hardship, race/ethnicity, education, age | x | distrust of vaccine and government, side effects | x | **People with low income,** being of any other **race than white, having financial hardship and low education** are less likely to get the vaccine. The main reasons are distrust in vaccine and government as well as fear of side effects. | x | x | vaccination |
| (14) | Baack, Brittney N. | panel household, survey | U.S. population | income, education, ethnicity | information exposure, relevant information | knowledge, attitude | x | **Lower education, lower income, and being black** is significantly associated with the lower likeliness of getting vaccinated. Reasons were not having enough information available about safety and effectiveness, lack of trust, knowledge, and a negative attitude. | x | x | getting vaccinated |
| (15) | Balasuriya, Lilanthi | cross-sectional qualitative, focus groups | Black and Latinx in New Heaven, U.S. | Black, Latinx, age, employment | information access, relevant information | knowledge, trust | x | **Black and Latinx** communities mentioned lack of trusted information, consistent messages, seeing diversity and fact-based information in wrong knowledge (e.g., feeling that vaccine accounts for deaths in African Americans), and mistrust (historically and experiences) as barriers to getting the vaccine. | x | x | getting vaccinated |
| (16) | Brown Wilson, Josiah | cross-sectional mixed methods survey (online and telephone) | Liberian population | education | x | knowledge | x | **Lower education i**s significantly associated with less knowledge and worse practice. | x | x | preventive behavior |
| (17) | Nakhostin-Ansari, Amin | cross-sectional online survey | Iranian population | occupation, education, age | x | knowledge, attitude | x | **Low education** is significantly associated with lower knowledge and less preventive behavior. | x | x | preventive behavior |
| (18) | Alsan, Marcella | cross-sectional online survey | U.S. population | age, education, occupation, race/ethnicity, income | x | knowledge | **People with low income** have significantly less knowledge of transmission and of symptoms. | **African American**s have significantly less knowledge of transmission and of symptoms. They are more likely to report being infected than white Americans. They are also more likely to leave home frequently. | x | x | being infected, preventive behavior |
| (19) | Vicerra, Paolo Miguel Manalang | cross-sectional, online survey | older Thai population (60+) | age (education) | x | knowledge | x | Only 43% of **older adults** have proper knowledge, and only 33% adopted preventive behavior. | x | x | behavior |
| (20) | Cervantes, Lilia | cross-sectional qualitative telephone interviews | Latinx hospitalized COVID-19 patients in the U.S. | Latinx (ethnic minority), income, education, age, employment | social media as an information source | misinformation, disbelief, trust | x | **Latinx** hospitalized covid 19 patients name misinformation, disbelief due to social media as the main source of information, and social norms, among other reasons for being infected. | x | x | hospitali-zation |
| (21) | Gautam, Vaishali | cross-sectional telephone survey | chronic disease patients in Rajasthan | chronic disease | health literacy | awareness of preventive measures | x | People with **chronic diseases** report low health literacy (65.8%) and low preventive behavior (45.1%). Low health literacy is associated with low awareness and lower preventive behavior. | x | x | preventive behavior |
| (22) | Devkota, Hridaya Raj | cross-sectional semi-structured telephone survey | Nepalese population | education, age, ethnicity | x | knowledge, attitude | x | **Low education** is significantly associated with low knowledge, low attitude, and low practice. | x | x | preventive behavior |
| (23) | Bazaid, Abdulrahman S. | cross-sectional, online social media survey | Saudi Arabian population | age, education, income | x | knowledge | **Low education** is significantly associated with low knowledge. | **Low income** is significantly associated with low knowledge and low practice. | x | x | preventive behavior |
| (24) | Siddiqui, Ammar Ahmed | cross-sectional online survey | Saudi Arabian population | age, education | x | knowledge | x | **Lower education** was significantly related to lower knowledge in some questions and lower preventive measures. | x | x | preventive behavior |
| (25) | Ayele, Alemu Degu | cross-sectional, face-to-face survey (structured) | pregnant women in Ethiopia | education, age. occupation | x | knowledge | Pregnant women **who were not occupied** were significantly less knowledgeable. | Pregnant women with **lower education** were less knowledgeable and showed significantly lower preventive practice. | x | x | preventive behavior |
| (26) | Kumbeni, Maxwell Tii | cross-sectional, face-to-face survey (structured) | pregnant women who accessed antenatal care services in the Nabdam District in Ghana. | age, education, chronic disease | x | knowledge | x | **Low education** is significantly related to lower COVID-19 knowledge and less preventive behavior. | x | People living with chronic conditions have significantly better preventive behavior. | preventive behavior |
| (27) | Kasemy, Zeinab A. | cross-sectional survey (online & personal) | Egyptian population | education, occupation-al status, age, income | x | knowledge, attitude | **Low income** is significantly associated with a negative attitude. | **Low education** was significantly associated with poor knowledge and practice. **Unemployed** showed more negative attitude and low practice. | **Low income**: good knowledge but poor practice! | x | preventive behavior |
| (28) | Ipsen, Catherine | cross-sectional, online survey | people with disabilities in the U.S. | disabilities (chronic condition), age, education, ethnicity | x | trust in official COVID-19 information sources | x | **People with communication disabilities** report lower trust in official information sources and lower practice than those with other disabilities. | x | x | preventive behavior |
| (29) | Christy, Josephine S. | cross-sectional, telephone survey | Indian population | education | x | knowledge, attitude | x | **Low education** is significantly associated with low attitude, knowledge, and practice. | x | x | preventive behavior |
| (30) | Ferdous, Most Zannatul | cross-sectional, online survey | Bangladeshi population | education, occupation, family income | x | knowledge, attitude | **Being unemployed** is significant with a more negative attitude. | **Low education i**s significantly associated wit**h l**ow knowledge, negative attitude, and low preventive behavior. **Low income** is significantly associated with negative attitudes and ow preventive behavior. | x | x | preventive behavior |
| (31) | Islam, Saiful | cross-sectional, face-to-face survey | Bangladeshi population living in slums | low income, poor neighborhood, age, education | x | knowledge, attitude | x | People living in slums in Bangladesh **(neighborhood)** generally have low knowledge (35.% good)) and practice (97.5% not using hand sanitizer). | x | x | preventive behavior |
| (32) | Lau, Lincoln Leehang | cross-sectional face-to-face survey | low-income population in the Philippines | age, education, income | x | knowledge, attitude | x | **Low-income population** in the Philippines shows low knowledge about measures (32.4% social distancing, 40% avoiding crowds), and practice is generally low (32.4 social distancing, 40% avoiding crowds, 28% mask-wearing). | x | x | preventive behavior |
| (33) | Takoudjou Dzomo, Guy Rodrigue | cross-sectional, face-to-face survey | Chadian population | age, level of education, occupation | x | knowledge, attitude | x | **Low education** is significantly associated with lower knowledge and practice. | x | x | preventive behavior |
| (34) | Srichan, Peeradone | cross-sectional, face-to-face survey | Thai population | age, education, income, nationality, chronic disease | x | knowledge, attitude | x | **Low education** was significantly associated with lower knowledge and lower preventive behavior. | **People of older age** have significantly lower preparedness. | x | preventive behavior (preparedness) |
| (35) | Yoseph, Amanuel | cross-sectional, face-to-face survey | South Ethiopian population | age, ethnicity, monthly income quintiles, educational levels | x | knowledge, attitude | **Low education** is significantly associated with a negative attitude. | **Low education and low income** are significantly associated with lower knowledge and lower preventive practices. | x | x | preventive behavior |
| (36) | Honarvar, Behnam | cross-sectional, face-to-face survey | Iranian population | age, education, occupation | x | knowledge | x | **Older people and low educated people** had significantly lower knowledge and lower practice. | x | x | preventive behavior |
| (37) | Serwaa, Dorcas | cross-sectional, online survey | Ghanaian population | education, employment | x | knowledge, risk perceptions | **Unemployment** is significantly associated with lower knowledge. | **Low education** is significantly associated with lower knowledge and preventive behavior. | x | x | preventive behavior (preparedness) |
| (38) | Alkhaldi, Ghadah | cross-sectional online survey | Saudi Arabian population | education, age, income, chronic disease, occupation, migration | x | risk perception | x | **Low income is** significantly associated with lower perceived severity and susceptibility as well as lower adoption of preventive behavior. | **Migrants, unemployed and old age** have significantly lower practice. | x | preventive behavior |
| (39) | Saqlain, Muhammad | cross-sectional, online survey | Pakistani population | income, education, employment status | x | knowledge | **Unemployment** is significantly associated with lower knowledge. | **Low income and low education** are significantly associated with lower knowledge and less preventive practice. | x | x | preventive behavior |
| (40) | Guo, Ziqiu | cross-sectional survey (telephone and web) | Hong Kong (Chinese) population | SES (education and income), age, employment status, chronic disease | seeking web-based information, e-health literacy |  | **Chronic disease** is significantly associated with less seeking web-based information. **Old age** is significantly associated with less seeking web-based information and low e-health literacy. | **Low SES (income and education)** is significantly associated with lower e-health, literacy, less seeking behavior, and less preventive behavior. | x | x | preventive behavior |
| (41) | Chandler, Rasheeta | cross-sectional, qualitative interviews | Black women living in the U.S. | employment, education, income | sources of information | confusion | x | **(79%) of Black women** are confused by the information they receive or find about covid-19, and they experience mental health problems, such as anxiety in combination with other implications the pandemic has on their lives. | x | x | mental health |
| (42) | Han, Bingfeng | cross-sectional, online survey | Chinese population | education | x | knowledge | x | **Low education** is significantly associated with less knowledge and lower practice scores. | x | x | preventive behavior |
| (43) | Gao, Huiming | cross-sectional online survey | Chinese population | age, education, occupation | x | knowledge | x | **Low education** is significantly associated with low knowledge and less practice. | x | x | preventive behavior |
| (44) | Alhazmi, Amani | cross-sectional online survey | Saudi Arabian population | age, nationality, occupation, education | x | knowledge, attitude | x | **Low education** is significantly associated with low knowledge and less practice. | x | Non-Saudis have better knowledge than Saudis. | preventive behavior |
| (45) | Afzal, Muhammad Sohail | cross-sectional, online survey | Pakistani population | education, occupation, income | x | knowledge | **Low education** is significantly associated with a lower attitude. | **Low income** is significantly associated with lower knowledge and attitude and lower practice. | x | x | preventive behavior |
| (46) | Addis, Sisay Gedamu | cross-sectional, face-to-face survey (structured) | people with chronic diseases in Ethiopia | chronic disease, education, age, occupation | x | knowledge, attitude | Patients with **chronic diseases** showed generally low knowledge (34.6% good) | x | x | Patients with chronic diseases have high preventive practice (>80%) | preventive behavior |
| (47) | Feleke, Bekele Taye | cross-sectional, face-to-face survey | Ethiopian outpatient patients | education/ ability to read and write | x | knowledge, attitude | **Low education** (unable to read and write) is significantly associated with a more negative attitude and less knowledge. | x | x | x | preventive behavior |
| (48) | Mousa, Khawla Nasr Aldeen Altayb | cross-sectional online survey | Sudanese population | age, education | x | knowledge | **Low education** is significantly associated with low knowledge. | x | x | x | preventive behavior |
| (49) | Mahoney, Dillon | cross-sectional qualitative interviews | resettled refugees from Congo in Tampa (U.S.) | refugees | Understand-ing information (English proficiency), critical health literacy | knowledge | **Refugees** have high knowledge but low trust in hospitals and low critical health literacy | x | x | Refugees have high knowledge, high social distancing, and testing. | testing behavior, preventive behavior |
| (50) | Irigoyen-Camacho, Maria Esther | cross-sectional, telephone survey | older Mexican adults (65+) | income, education in aged | x | risk perception | Low perceived susceptibility (54.2%) and severity (33.4%) were found in **older adults.** | x | x | 57.6% of older adults decided to stay at home. To 15.1%, this is mediated by perceived severity. | behavior |
| (51) | Murugan-andam, Partheeban | cross-sectional telephone survey | people with severe mental illness (SMI) in India | chronic condition (SMI) | x | awareness, knowledge | **2/3 of people with SMI** are not well aware and knowledgeable about COVID-19, but only 30% have relapses | x | x | x | mental health |
| (52) | Van Nhu, Ha | cross-sectional, online survey | Vietnamese population | education | x | knowledge, attitude towards preventive measures | **Low education** is significantly associated with poorer knowledge. **Higher age i**s significantly associated with a poorer attitude. | x | x | x | preventive behavior |
| (53) | Erdem, Dilek | cross-sectional, survey, telephone and face to face | oncology patients in Turkey | chronic disease (cancer patients), low education | x | awareness of risks | **People with chronic disease** show is significantly lower knowledge (2/3) | x | x | Living with a chronic disease is significantly associated with high preventive behavior. | preventive behavior |
| (54) | Tsai, Feng-Jen | cross-sectional, telephone survey | Taiwanese population | education, employment status, age | x | risk perception, vaccine knowledge, vaccination intention | People of **older age** show a significantly higher vaccination hesitancy. | x | x | Low education and unemployment are significantly associated with higher willingness to get the vaccine than high educated and high income. | vaccination |
| (55) | Marendić, Mario | cross-sectional. social media survey | Croatian population | education, age, employment status, | x | knowledge, attitude | **Low education** is significantly associated with lower knowledge. | x | x | Lower education is significantly associated with increased use of protective equipment (mask/gloves). | preventive measures |
| (56) | Bailey, Stacy Cooper | longitudinal telephone survey | U.S. population | ethnicity, poverty level, occupation, age, English proficiency, chronic condition | health literacy | awareness of risk, knowledge, | Significantly lower risk awareness is found in **Black people** | x | x | x | preventive behavior |
| (57) | Rattay, Petra | cross-sectional online survey | German population | education | x | risk perception, knowledge | **Low education** is significantly associated with lower knowledge. | x | x | x | preventive behavior |
| (58) | Belete, Zebader Walle | cross-sectional, face-to-face survey | Ethiopian hospital visitors | occupation, education, chronic disease, income | source of information | knowledge, attitude | **Low education** is significant with low knowledge and negative attitude. | x | x | x | preventive behavior |
| (59) | Qutob, Nouar | cross-sectional, telephone survey | Palestinian population | education, employment | x | awareness, knowledge, attitude | Low awareness is significantly associated with **low education.** | x | x | x | preventive behavior |
| (60) | Yue, Shaoting | cross-sectional, online survey | the population of the Henan Province (China) | age, educational level | x | knowledge, attitude | **Low education** is significantly associated with lower knowledge | x | x | x | preventive behavior |
| (61) | Hezima, Ahmed | cross-sectional, online survey | Sudanese population | age, education | x | knowledge, attitude | **Low education and older age** were significant with low knowledge and high age with a negative attitude. | x | x | People of older age show significantly better preventive practice than those of young age. | preventive behavior |
| (62) | Baig, Mukhtiar | cross-sectional online survey | Saudi Arabian population | education, age, migrant status, | x | attitude, knowledge, misconceptions | **Low education and older age** are significantly associated with less knowledge. | x | x | People of older age show high preventive behavior. | preventive behavior |
| (63) | Chang, Chee Tao | cross-sectional, online survey | Malaysian population | education, income, ethnicity, occupation, chronic illness | x | knowledge about covid | **Low education and low income** are significantly associated with lower knowledge. | x | x | People with low income show significantly better practice than those with high income. | preventive behavior |
| (64) | Alobuia, Wilson M. | cross-sectional, telephone survey | U.S. population | age, education, income, ethnicity, chronic illness | x | knowledge | **Being Black, having lower income, and having lower education** are significantly associated with lower knowledge. **Being black and low educated** are significantly associated with lower attitude, | x | x | Being Black is significantly associated with a better practice score. | preventive behavior |
| (65) | Wang, Xiaomin | cross-sectional, survey | Chinese population | ethnicity, education, income. | exposure to risk messages, exposure to misinformation | risk perception, belief in misinformation | **Low education and low income are significantly associated with less exposure to preventive messages, correction of misinformation, and** firmer belief in misinformation. | x | **Ethnic minorities** are more exposed to misinformation correction and have a high level of risk perception but a low level of adopting the behavior. | x | preventive measures |
| (66) | Chan, Emily Ying Yang | cross-sectional, telephone survey | Hong Kong (Chinese) population | age, education, income | x | risk perception, knowledge | **Low education** is significantly associated with less knowledge. | x | x | x | preventive measures |
| (67) | Adesegun, Oluwaseyitan A. | cross-sectional online survey | Nigerian population | age, education, ethnicity, occupation | source of information | attitude, knowledge | **Unemployed people** have a significantly lower knowledge and attitude than employed people. | x | x | x | preventive behavior |
| (68) | Azlan, Arina Anis | cross-sectional online survey | Malaysian population | age, income, occupation | x | knowledge, attitude | **Low income** is significantly associated with less knowledge. | x | **Older adults** have more knowledge but have significant lower practice | People with lower income show significantly higher practice than those with high income. | preventive behavior |
| (69) | Bates, Benjamin R. | cross-sectional, online survey | Ecuadorian population | age, education, occupation | x | knowledge | **Unemployed people** have a significantly lower knowledge than employed people | x | x | Unemployment is significantly associated with high hand washing. | preventive behavior |
| (70) | Al-Hanawi, Mohammed K. | cross-sectional online survey | Saudi Arabian population | age, education, occupation, income | x | knowledge | **Older age, low education, and low income** are significantly associated with lower knowledge. | x | x | x | preventive behavior |
| (71) | El-Masry, Eman A. | cross-sectional, online survey | Saudi Arabian population | age, income, education, chronic disease | source of information | knowledge | x | x | **Older age** is significantly associated with lower preventive behavior. | x | preventive behavior |
| (72) | Mouchtouri, Varvara A. | cross-sectional, telephone survey | Greek population | occupation, education, income, age | x | knowledge | x | x | **Low education** is significantly associated with low preventive behavior. | x | preventive behavior |
| (73) | Tavares, Darlene Mara Dos Santos | cross-sectional, telephone survey | older Brazilian adults (60+) | age (education, higher age) | x | knowledge | x | x | High knowledge was found in 86.6- 90.8% of **older adults**- but many could not implement all preventive behaviors. | x | preventive behavior |
| (74) | O'Conor, Rachel | cross-sectional, telephone survey | older people with chronic health conditions in the U.S. | age, ethnicity, occupation, income, chronic condition | health literacy | knowledge | x | x | **Older people** have good knowledge (71%), but only 38% report preventive practice. | x | preventive behavior |
| (75) | Brankston, Gabrielle | cross-sectional, online survey | Canadian population | age, income, education, employment status | x | risk perception, attitude | x | x | **Low education** is significantly associated with less mask usage. | Unemployment is associated with higher than employment. | preventive measures |
| (76) | Nwonwu, Elizabeth Uzoamaka | cross-sectional, semi-structured survey | Nigerian population, state of Enugu | education, age | x | knowledge | x | x | x | Low education is associated with higher preventive behavior than high education. | preventive behavior |
| (77) | Yodmai, Korravarn | cross-sectional face-to-face survey | older Thai population (60+) | age, income, education | health literacy. access to health information |  | x | x | x | 58.2% of older adults have access to information. Older adults have good preventive behavior (72.6%). | preventive behavior |
| (78) | Saeed, Shazina | cross-sectional, online survey | Indians with comorbidities | chronic disease, education, occupation, age, income | x | attitude, knowledge | x | x | x | People with chronic diseases have good knowledge (82.7%) and attitude (75.0%). | preventive behavior |
| (79) | Akalu, Yonas | cross-sectional, face-to-face survey | chronic disease patients in Ethiopia | chronic disease, age, education, income | x | knowledge, attitude | x | x | x | Good knowledge was found in people with chronic disease (33.9% insufficient), and 47.3% have poor practice (47.3%) | preventive behavior |
| (80) | Wolf, Michael S. | cross sectional, telephone survey | people with chronic conditions in the U.S. | ethnicity, aged, income, chronic conditions, limited English proficiency (LEP) | x | awareness of risk, knowledge, attitude, | x | x | x | In people with chronic diseases only few lack of critical knowledge (28.3% - 30.2%). They have a rather low risk perception (24.6%) and not many do not change their behavior due to the pandemic (21.9%) | preventive measures |
| (81) | Alaloul, Fawwaz | cross-sectional, online survey | people living in a Gulf country | income and education, age, chronic disease | x | knowledge | x | x | x | Peoplpe with chronic diseases show significantly better preventive practice than people without chronic diseases. | preventive measures |
| (82) | Ceccato, Irene | cross-sectional online survey | Italian population | older age, education | x | attitude, fear of COVID | x | x | x | People of older age show significantly higher risk perception. | preventive behaviors |
| (83) | Bui, Ha Thi Thu | cross-sectional, survey | migrant workers in Vietnam | migrant (occupation, sex, age, education, ethnicity) | information exposure | knowledge | x | x | x | Migrants have good knowledge about preventive measures (>90%), are exposed to information, and have good practices (81.1%). | preventive behavior |
| (84) | Luk, Tzu Tsun | cross-sectional, telephone survey | Hong Kong population | education, age | exposure to misinformation on social media |  | x | x | x | Low-educated and older people are less exposed to misinformation regarding alcohol and tobacco consumption that may prevent covid 19. Exposure to these claims was associated with increased alcohol and tobacco consumption. | health behavior (alcohol and tobacco consumption) |
| (85) | Iradukunda, Patrick Gad | cross-sectional, face-to-face survey | Rwanda, HIV community | HIV, age, occupational status | x | knowledge, attitude | x | x | x | People living with HIV show high knowledge (97%), a positive attitude (74%), and good preventive practice is (90%). | preventive behavior |
| (86) | Pal, Rimesh | cross-sectional, online survey | Indian population with diabetes mellitus | chronic disease, age, education, occupation | x | knowledge, attitude, risk-perception | x | x | x | Average knowledge (74%), high-risk perception (88%), positive attitude regarding self-protection (98%), and good hygiene practice (100%) were found among Indian people with diabetes. No differences in sociodemographic characteristics were found. | preventive behavior |
| (87) | Chen, Ying | cross-sectional, online survey | elderly Chinese population (60+) | age, education, income | x | knowledge | x | x | x | Good knowledge (87% correct) and behavior (>70%) were found in people of older age in China. Knowledge and behavior are significantly associated. | preventive behavior |
| (88) | Defar, Atkure | cross-sectional, face-to-face survey | highly exposed Ethiopian population | income, age | x | knowledge | x | x | x | Older age and low income are significantly associated with the better preventive practice. | preventive behavior (preparedness) |
| (89) | Luo, Yunjuan | cross-sectional, online survey | U.S. population | age, ethnicity, education, income, | x | perceived severity | x | x | x | Older people have significantly higher perceived severity and apply more preventive behavior than younger people. | preventive behavior |
| (90) | Paul, Alak | cross-sectional online survey | Bangladeshi | education, gender, age, occupation | x | knowledge, attitude towards preventive measures | x | x | x | No vulnerable variable was significant with knowledge, attitude, and behavior | preventive behavior |
| (91) | Williams, Lovoria B. | cross-sectional online survey | Black Kentucky and white Appalachian church population in U.S. | ethnicity, age, education, income | x | intention to practice behavior and vaccine hesitancy | x | x | x | No significant differences were found between Black and white people in terms of knowledge and mental health. | mental health |
| (92) | Maher, Paul J. | longitudinal, online survey | UK population | income, education, perceived SES | x | attitude, skepticism towards science | x | x | x | Attitude, skepticism, and behavior are not significantly related to any vulnerable variable. | preventive behavior |

# References

1. Rhodes SD, Mann-Jackson L, Alonzo J, Garcia M, Tanner AE, Smart BD, et al. A rapid qualitative assessment of the impact of the COVID-19 pandemic on a racially/ethnically diverse sample of gay, bisexual, and other men who have sex with men living with HIV in the US south. AIDS Behav. 2021;25(1):58–67.

2. Okello G, Izudi J, Teguzirigwa S, Kakinda A, Van Hal G. Findings of a cross-sectional survey on knowledge, attitudes, and practices about COVID-19 in Uganda: implications for public health prevention and control measures. Biomed Res Int. 2020;2020:5917378.

3. Clements JM. Knowledge and behaviors toward COVID-19 among US residents during the early days of the pandemic: Cross-sectional online questionnaire. JMIR Public Health Surveill. 2020;6(2):e19161.

4. Xu N, Zhang Y, Zhang X, Zhang G, Guo Z, Zhao N, et al. Knowledge, attitudes, and practices of urban residents toward COVID-19 in Shaanxi during the post-lockdown period. Front Public Health. 2021;9:659797.

5. Fatmi Z, Mahmood S, Hameed W, Qazi I, Siddiqui M, Dhanwani A, et al. Knowledge, attitudes and practices towards COVID-19 among Pakistani residents: information access and low literacy vulnerabilities. East Mediterr Health J. 2020;26(12):1446–55.

6. Alnasser AHA, Al-Tawfiq JA, Al-Kalif MSH, Shahadah RFB, Almuqati KSA, Al-Sulaiman BSA, et al. Public knowledge, attitudes, and practice towards COVID-19 pandemic in Saudi Arabia: A web-based cross-sectional survey. Med Sci (Basel). 2021;9(1):11.

7. Alsaif B, Elhassan NEE, Itumalla R, Ali KE, Alzain MA. Assessing the level of awareness of COVID-19 and prevalence of general anxiety disorder among the Hail community, Kingdom of Saudi Arabia. Int J Environ Res Public Health. 2021;18(13):7035.

8. Al Zabadi H, Yaseen N, Alhroub T, Haj-Yahya M. Assessment of quarantine understanding and adherence to lockdown measures during the COVID-19 pandemic in Palestine: Community experience and evidence for action. Front Public Health. 2021;9:570242.

9. Jimenez ME, Rivera-Núñez Z, Crabtree BF, Hill D, Pellerano MB, Devance D, et al. Black and Latinx community perspectives on COVID-19 mitigation behaviors, testing, and vaccines. JAMA Netw Open. 2021;4(7):e2117074.

10. Rahman FN, Bhuiyan MAA, Hossen K, Khan HTA, Rahman AF, Dalal K. Challenges in preventive practices and risk communication towards COVID-19: A cross-sectional study in Bangladesh. Int J Environ Res Public Health. 2021;18(17):9259.

11. Ko NY, Lu WH, Chen YL, Li DJ, Chang YP, Wang PW, et al. Cognitive, affective, and behavioral constructs of COVID-19 health beliefs: A comparison between sexual minority and heterosexual individuals in Taiwan. Int J Environ Res Public Health. 2020;17(12):4282.

12. Sengeh P, Jalloh MB, Webber N, Ngobeh I, Samba T, Thomas H, et al. Community knowledge, perceptions and practices around COVID-19 in Sierra Leone: A nationwide, cross-sectional survey. BMJ Open. 2020 Sep 17;10(9):e040328.

13. Nguyen KH, Nguyen K, Corlin L, Allen JD, Chung M. Changes in COVID-19 vaccination receipt and intention to vaccinate by socioeconomic characteristics and geographic area, United States, January 6 – March 29, 2021. Ann Med. 2021;53(1):1419–28.

14. Baack BN, Abad N, Yankey D, Kahn KE, Razzaghi H, Brookmeyer K, et al. COVID-19 vaccination coverage and intent among adults aged 18-39 Years - United States, March-May 2021. MMWR Morb Mortal Wkly Rep. 2021;70(25):928–33.

15. Balasuriya L, Santilli A, Morone J, Ainooson J, Roy B, Njoku A, et al. COVID-19 vaccine acceptance and access among Black and Latinx communities. JAMA Netw Open. 2021;4(10):e2128575.

16. Brown Wilson J, Deckert A, Shah R, Kyei N, Copeland Dahn L, Doe-Rogers R, et al. COVID-19-related knowledge, attitudes and practices: a mixed-mode cross-sectional survey in Liberia. BMJ Open. 2021;11(7):e049494.

17. Nakhostin-Ansari A, Aghajani F, Khonji MS, Aghajani R, Pirayandeh P, Allahbeigi R, et al. Did Iranians respect health measures during Nowruz holidays? A study on Iranians’ knowledge, attitude and practice toward COVID-19. J Prev Med Hyg. 2020;61(4):E501–7.

18. Alsan M, Stantcheva S, Yang D, Cutler D. Disparities in coronavirus 2019 reported incidence, knowledge, and behavior among US adults. JAMA Netw Open. 2020;3(6):e2012403.

19. Vicerra PMM. Disparity between knowledge and practice regarding COVID-19 in Thailand: A cross-sectional study of older adults. PLoS One. 2021;16(10):e0259154.

20. Cervantes L, Martin M, Frank MG, Farfan JF, Kearns M, Rubio LA, et al. Experiences of Latinx individuals hospitalized for COVID-19: A qualitative study. JAMA Netw Open. 2021;4(3):e210684.

21. Gautam V, S D, Rustagi N, Mittal A, Patel M, Shafi S, et al. Health literacy, preventive COVID 19 behaviour and adherence to chronic disease treatment during lockdown among patients registered at primary health facility in urban Jodhpur, Rajasthan. Diabetes Metab Syndr. 2021;15(1):205–11.

22. Devkota HR, Sijali TR, Bogati R, Clarke A, Adhikary P, Karkee R. How does public knowledge, attitudes, and behaviors correlate in relation to COVID-19? A community-based cross-sectional study in Nepal. Front Public Health. 2020;8:589372.

23. Bazaid AS, Aldarhami A, Binsaleh NK, Sherwani S, Althomali OW. Knowledge and practice of personal protective measures during the COVID-19 pandemic: A cross-sectional study in Saudi Arabia. PLoS One. 2020;15(12):e0243695.

24. Siddiqui AA, Alshammary F, Amin J, Rathore HA, Hassan I, Ilyas M, et al. Knowledge and practice regarding prevention of COVID-19 among the Saudi Arabian population. Work. 2020;66(4):767–75.

25. Ayele AD, Mihretie GN, Belay HG, Teffera AG, Kassa BG, Amsalu BT. Knowledge and practice to prevent COVID-19 and its associated factors among pregnant women in Debre Tabor Town Northwest Ethiopia, a community-based cross-sectional study. BMC Pregnancy Childbirth. 2021;21(1):397.

26. Kumbeni MT, Apanga PA, Yeboah EO, Lettor IBK. Knowledge and preventive practices towards COVID-19 among pregnant women seeking antenatal services in Northern Ghana. PLoS One. 2021;16(6):e0253446.

27. Kasemy ZA, Bahbah WA, Zewain SK, Haggag MG, Alkalash SH, Zahran E, et al. Knowledge, attitude and practice toward COVID-19 among Egyptians. J Epidemiol Glob Health. 2020;10(4):378–85.

28. Ipsen C, Myers A, Sage R. A cross-sectional analysis of trust of information and COVID-19 preventative practices among people with disabilities. Disability and Health Journal. 2021;14(2):101062.

29. Christy JS, Kaur K, Gurnani B, Hess OM, Narendran K, Venugopal A, et al. Knowledge, attitude and practise toward COVID-19 among patients presenting to five tertiary eye care hospitals in South India - A multicentre questionnaire-based survey. Indian J Ophthalmol. 2020;68(11):2385–90.

30. Ferdous MZ, Islam MS, Sikder MT, Mosaddek ASM, Zegarra-Valdivia JA, Gozal D. Knowledge, attitude, and practice regarding COVID-19 outbreak in Bangladesh: An online-based cross-sectional study. PLoS One. 2020;15(10):e0239254.

31. Islam S, Emran GI, Rahman E, Banik R, Sikder T, Smith L, et al. Knowledge, attitudes and practices associated with the COVID-19 among slum dwellers resided in Dhaka City: A Bangladeshi interview-based survey. J Public Health (Oxf). 2021;43(1):13–25.

32. Lau LL, Hung N, Go DJ, Ferma J, Choi M, Dodd W, et al. Knowledge, attitudes and practices of COVID-19 among income-poor households in the Philippines: A cross-sectional study. J Glob Health. 2020;10(1):011007.

33. Takoudjou Dzomo GR, Bernales M, López R, Djofang Kamga Y, Kila Roskem JP, Deassal Mondjimbaye F, et al. Knowledge, attitudes and practices regarding COVID‑19 in N’Djamena, Chad. J Community Health. 2021;46(2):259–66.

34. Srichan P, Apidechkul T, Tamornpark R, Yeemard F, Khunthason S, Kitchanapaiboon S, et al. Knowledge, attitudes and preparedness to respond to COVID-19 among the border population of northern Thailand in the early period of the pandemic: A cross-sectional study. WHO South-East Asia J Public Health. 2020;9(2):118–25.

35. Yoseph A, Tamiso A, Ejeso A. Knowledge, attitudes, and practices related to COVID-19 pandemic among adult population in Sidama Regional State, Southern Ethiopia: A community based cross-sectional study. PLoS One. 2021;16(1):e0246283.

36. Honarvar B, Lankarani KB, Kharmandar A, Shaygani F, Zahedroozgar M, Rahmanian Haghighi MR, et al. Knowledge, attitudes, risk perceptions, and practices of adults toward COVID-19: a population and field-based study from Iran. Int J Public Health. 2020;65(6):731–9.

37. Serwaa D, Lamptey E, Appiah AB, Senkyire EK, Ameyaw JK. Knowledge, risk perception and preparedness towards coronavirus disease-2019 (COVID-19) outbreak among Ghanaians: A quick online cross-sectional survey. Pan Afr Med J. 2020;35(Suppl 2):44.

38. Alkhaldi G, Aljuraiban GS, Alhurishi S, De Souza R, Lamahewa K, Lau R, et al. Perceptions towards COVID-19 and adoption of preventive measures among the public in Saudi Arabia: A cross sectional study. BMC Public Health. 2021;21(1):1251.

39. Saqlain M, Ahmed A, Nabi I, Gulzar A, Naz S, Munir MM, et al. Public knowledge and practices regarding coronavirus disease 2019: A cross-sectional survey from Pakistan. Front Public Health. 2021;9:629015.

40. Guo Z, Zhao SZ, Guo N, Wu Y, Weng X, Wong JYH, et al. Socioeconomic disparities in ehealth literacy and preventive behaviors during the COVID-19 pandemic in Hong Kong: Cross-sectional study. J Med Internet Res. 2021;23(4):e24577.

41. Chandler R, Guillaume D, Parker AG, Mack A, Hamilton J, Dorsey J, et al. The impact of COVID-19 among Black women: Evaluating perspectives and sources of information. Ethn Health. 2021;26(1):80–93.

42. Han B, Zhao T, Liu B, Liu H, Zheng H, Wan Y, et al. Public awareness, individual prevention practice, and psychological effect at the beginning of the COVID-19 outbreak in China. Journal of Epidemiology. 2020;30(10):474–82.

43. Gao H, Hu R, Yin L, Yuan X, Tang H, Luo L, et al. Knowledge, attitudes and practices of the Chinese public with respect to coronavirus disease (COVID-19): An online cross-sectional survey. BMC Public Health. 2020 Nov 30;20(1):1816.

44. Alhazmi A, Ali MHM, Mohieldin A, Aziz F, Osman OB, Ahmed WA. Knowledge, attitudes and practices among people in Saudi Arabia regarding COVID-19: A cross-sectional study. Journal of Public Health Research. 2020 Sep 18;9(3):1867.

45. Afzal MS, Khan A, Qureshi UUR, Saleem S, Saqib MAN, Shabbir RMK, et al. Community-based assessment of knowledge, attitude, practices and risk factors regarding COVID-19 among Pakistanis residents during a recent outbreak: A cross-sectional survey. J Community Health. 2021;46(3):476–86.

46. Addis SG, Nega AD, Miretu DG. Knowledge, attitude and practice of patients with chronic diseases towards COVID-19 pandemic in Dessie town hospitals, Northeast Ethiopia. Diabetes Metab Syndr. 2021;15(3):847–56.

47. Feleke BT, Wale MZ, Yirsaw MT. Knowledge, attitude and preventive practice towards COVID-19 and associated factors among outpatient service visitors at Debre Markos compressive specialized hospital, north-west Ethiopia, 2020. PLoS One. 2021;16(7):e0251708.

48. Mousa KNAA, Saad MMY, Abdelghafor MTB. Knowledge, attitudes, and practices surrounding COVID-19 among Sudan citizens during the pandemic: An online cross-sectional study. Sudan Journal of Medical Sciences (SJMS). 2020;15(2):32–45.

49. Mahoney D, Obure R, Billingsley K, Inks M, Umurutasate E, Baer RD. Evaluating understandings of State and Federal pandemic policies: The situation of refugees from the Congo wars in Tampa, Florida. Human Organization. 2020;79(4):271–80.

50. Irigoyen-Camacho ME, Velazquez-Alva MC, Zepeda-Zepeda MA, Cabrer-Rosales MF, Lazarevich I, Castaño-Seiquer A. Effect of income level and perception of susceptibility and severity of COVID-19 on stay-at-home preventive behavior in a group of older adults in Mexico City. Int J Environ Res Public Health. 2020;17(20):E7418.

51. Muruganandam P, Neelamegam S, Menon V, Alexander J, Chaturvedi SK. COVID-19 and severe mental illness: Impact on patients and its relation with their awareness about COVID-19. Psychiatry Res. 2020;291:113265.

52. Van Nhu H, Tuyet-Hanh TT, Van NTA, Linh TNQ, Tien TQ. Knowledge, Attitudes, and Practices of the Vietnamese as Key Factors in Controlling COVID-19. J Community Health. 2020;45(6):1263–9.

53. Erdem D, Karaman I. Awareness and perceptions related to COVID-19 among cancer patients: A survey in oncology department. Eur J Cancer Care (Engl). 2020;29(6):e13309.

54. Tsai FJ, Yang HW, Lin CP, Liu JZ. Acceptability of COVID-19 vaccines and protective behavior among adults in Taiwan: Associations between risk perception and willingness to vaccinate against COVID-19. Int J Environ Res Public Health. 2021;18(11):5579.

55. Marendić M, Bokan I, Buljan I, Dominiković P, Suton R, Kolčić I. Adherence to epidemiological measures and related knowledge and attitudes during the coronavirus disease 2019 epidemic in Croatia: A cross-sectional study. Croat Med J. 2020;61(6):508–17.

56. Bailey SC, Serper M, Opsasnick L, Persell SD, O’Conor R, Curtis LM, et al. Changes in COVID-19 knowledge, beliefs, behaviors, and preparedness among high-risk adults from the onset to the acceleration phase of the US outbreak. J Gen Intern Med. 2020;35(11):3285–92.

57. Rattay P, Michalski N, Domanska OM, Kaltwasser A, De Bock F, Wieler LH, et al. Differences in risk perception, knowledge and protective behaviour regarding COVID-19 by education level among women and men in Germany. Results from the COVID-19 Snapshot Monitoring (COSMO) study. PLoS One. 2021;16(5):e0251694.

58. Belete ZW, Berihun G, Keleb A, Ademas A, Berhanu L, Abebe M, et al. Knowledge, attitude, and preventive practices towards COVID-19 and associated factors among adult hospital visitors in South Gondar Zone Hospitals, Northwest Ethiopia. PLoS One. 2021;16(5):e0250145.

59. Qutob N, Awartani F. Knowledge, attitudes and practices (KAP) towards COVID-19 among Palestinians during the COVID-19 outbreak: A cross-sectional survey. PLoS One. 2021;16(1):e0244925.

60. Yue S, Zhang J, Cao M, Chen B. Knowledge, attitudes and practices of COVID-19 among urban and rural residents in China: A cross-sectional study. J Community Health. 2021;46(2):286–91.

61. Hezima A, Aljafari A, Aljafari A, Mohammad A, Adel I. Knowledge, attitudes, and practices of Sudanese residents towards COVID-19. East Mediterr Health J. 2020;26(6):646–51.

62. Baig M, Jameel T, Alzahrani SH, Mirza AA, Gazzaz ZJ, Ahmad T, et al. Predictors of misconceptions, knowledge, attitudes, and practices of COVID-19 pandemic among a sample of Saudi population. PLoS One. 2020 Dec 9;15(12):e0243526.

63. Chang CT, Lee M, Lee JCY, Lee NCT, Ng TY, Shafie AA, et al. Public KAP towards COVID-19 and antibiotics resistance: A Malaysian survey of knowledge and awareness. Int J Environ Res Public Health. 2021;18(8):3964.

64. Alobuia WM, Dalva-Baird NP, Forrester JD, Bendavid E, Bhattacharya J, Kebebew E. Racial disparities in knowledge, attitudes and practices related to COVID-19 in the USA. J Public Health (Oxf). 2020;42(3):470–8.

65. Wang C, Tian Q, Zhao P, Xiong M, Latkin CA, Gan Y, et al. Disease knowledge and attitudes during the COVID-19 epidemic among international migrants in China: a national cross-sectional study. Int J Biol Sci. 2020;16(15):2895–905.

66. Chan EYY, Huang Z, Lo ESK, Hung KKC, Wong ELY, Wong SYS. Sociodemographic predictors of health risk perception, attitude and behavior practices associated with health-emergency disaster risk management for biological hazards: The case of COVID-19 pandemic in Hong Kong, SAR China. Int J Environ Res Public Health. 2020;17(11):E3869.

67. Adesegun OA, Binuyo T, Adeyemi O, Ehioghae O, Rabor DF, Amusan O, et al. The COVID-19 crisis in Sub-Saharan Africa: Knowledge, attitudes, and practices of the Nigerian public. Am J Trop Med Hyg. 2020;103(5):1997–2004.

68. Azlan AA, Hamzah MR, Sern TJ, Ayub SH, Mohamad E. Public knowledge, attitudes and practices towards COVID-19: A cross-sectional study in Malaysia. PLOS ONE. 2020 May 21;15(5):e0233668.

69. Bates BR, Moncayo AL, Costales JA, Herrera-Cespedes CA, Grijalva MJ. Knowledge, attitudes, and practices towards COVID-19 among Ecuadorians during the outbreak: An online cross-sectional survey. J Community Health. 2020 Dec 1;45(6):1158–67.

70. Al-Hanawi MK, Angawi K, Alshareef N, Qattan AMN, Helmy HZ, Abudawood Y, et al. Knowledge, attitude and practice toward COVID-19 among the public in the Kingdom of Saudi Arabia: A cross-sectional study. Frontiers in Public Health. 2020;8:217.

71. El-Masry EA, Mohamed RA, Ali RI, Al Mulhim MF, Taha AE. Novel coronavirus disease-related knowledge, attitudes, and practices among the residents of Al-Jouf region in Saudi Arabia. J Infect Dev Ctries. 2021;15(1):32–9.

72. Mouchtouri VA, Agathagelidou E, Kofonikolas K, Rousou X, Dadouli K, Pinaka O, et al. Nationwide survey in Greece about knowledge, risk perceptions, and preventive behaviors for COVID-19 during the general lockdown in April 2020. Int J Environ Res Public Health. 2020;17(23):E8854.

73. Tavares DMDS, Oliveira NGN, Marchiori GF, Guimarães MSF, Santana LPM. Elderly individuals living by themselves: Knowledge and measures to prevent the novel coronavirus. Rev Lat Am Enfermagem. 2020;28:e3383.

74. O’Conor R, Opsasnick L, Benavente JY, Russell AM, Wismer G, Eifler M, et al. Knowledge and behaviors of adults with underlying health conditions during the onset of the COVID-19 U.S. outbreak: The Chicago COVID-19 comorbidities survey. J Community Health. 2020;45(6):1149–57.

75. Brankston G, Merkley E, Fisman DN, Tuite AR, Poljak Z, Loewen PJ, et al. Socio-demographic disparities in knowledge, practices, and ability to comply with COVID-19 public health measures in Canada. Can J Public Health. 2021;112(3):363–75.

76. Nwonwu EU, Ossai EN, Umeokonkwo CD, Ituma IB. Knowledge and preventive practice to COVID-19 among household heads in Enugu metropolis, South-East Nigeria. Pan Afr Med J. 2020;37:63.

77. Yodmai K, Pechrapa K, Kittipichai W, Charupoonpol P, Suksatan W. Factors associated with good COVID-19 preventive behaviors among older adults in urban communities in Thailand. J Prim Care Community Health. 2021;12:21501327211036252.

78. Saeed S, Awasthi AA, Nandi D, Kaur K, Hasan S, Janardhanan R. Knowledge, attitude and practice towards COVID-19 among individuals with associated comorbidities. J Med Life. 2021;14(2):225–37.

79. Akalu Y, Ayelign B, Molla MD. Knowledge, attitude and practice towards COVID-19 among chronic disease patients at Addis Zemen Hospital, Northwest Ethiopia. IDR. 2020;13:1949–60.

80. Wolf MS, Serper M, Opsasnick L, O’Conor RM, Curtis L, Benavente JY, et al. Awareness, attitudes, and actions related to COVID-19 among adults with chronic conditions at the onset of the U.S. outbreak: A cross-sectional survey. Ann Intern Med. 2020;173(2):100–9.

81. Alaloul F, Alomari K, Al Qadire M, Al-Dwaikat T. Public knowledge, attitude, practices, and level of anxiety toward the COVID-19 pandemic among people living in Oman. Nurs Forum. 2021;56(3):596–603.

82. Ceccato I, Palumbo R, Di Crosta A, La Malva P, Marchetti D, Maiella R, et al. Age-related differences in the perception of COVID-19 emergency during the Italian outbreak. Aging Ment Health. 2021;25(7):1305–13.

83. Bui HTT, Duong DM, Pham TQ, Mirzoev T, Bui ATM, La QN. COVID-19 stressors on migrant workers in Vietnam: Cumulative risk consideration. Int J Environ Res Public Health. 2021;18(16):8757.

84. Luk TT, Zhao S, Weng X, Wong JYH, Wu YS, Ho SY, et al. Exposure to health misinformation about COVID-19 and increased tobacco and alcohol use: A population-based survey in Hong Kong. Tob Control. 2021;30(6):696–9.

85. Iradukunda PG, Pierre G, Muhozi V, Denhere K, Dzinamarira T. Knowledge, attitude, and practice towards COVID-19 among people living with HIV/AIDS in Kigali, Rwanda. J Community Health. 2021;46(2):245–50.

86. Pal R, Yadav U, Grover S, Saboo B, Verma A, Bhadada SK. Knowledge, attitudes and practices towards COVID-19 among young adults with Type 1 Diabetes Mellitus amid the nationwide lockdown in India: A cross-sectional survey. Diabetes Res Clin Pract. 2020;166:108344.

87. Chen Y, Zhou R, Chen B, Chen H, Li Y, Chen Z, et al. Knowledge, perceived beliefs, and preventive behaviors related to COVID-19 among Chinese older adults: Cross-sectional web-based survey. J Med Internet Res. 2020;22(12):e23729.

88. Defar A, Molla G, Abdella S, Tessema M, Ahmed M, Tadele A, et al. Knowledge, practice and associated factors towards the prevention of COVID-19 among high-risk groups: A cross-sectional study in Addis Ababa, Ethiopia. PLoS One. 2021;16(3):e0248420.

89. Luo Y, Cheng Y, Sui M. The moderating effects of perceived severity on the generational gap in preventive behaviors during the COVID-19 pandemic in the U.S. Int J Environ Res Public Health. 2021;18(4):2011.

90. Paul A, Sikdar D, Hossain MM, Amin MR, Deeba F, Mahanta J, et al. Knowledge, attitudes, and practices toward the novel coronavirus among Bangladeshis: Implications for mitigation measures. PLoS One. 2020;15(9):e0238492.

91. Williams LB, Fernander AF, Azam T, Gomez ML, Kang J, Moody CL, et al. COVID-19 and the impact on rural and black church Congregants: Results of the C-M-C project. Res Nurs Health. 2021;44(5):767–75.

92. Maher PJ, MacCarron P, Quayle M. Mapping public health responses with attitude networks: The emergence of opinion-based groups in the UK’s early COVID-19 response phase. Br J Soc Psychol. 2020;59(3):641–52.
